# Antibacterial and Antibiofilm Activity of Selected Medicinal Plant Leaf Extracts Against Pathogens Implicated in Poultry Diseases

**DOI:** 10.3389/fvets.2022.820304

**Published:** 2022-03-02

**Authors:** Olasunkanmi S. Olawuwo, Ibukun M. Famuyide, Lyndy J. McGaw

**Affiliations:** Phytomedicine Programme, Department of Paraclinical Sciences, University of Pretoria, Pretoria, South Africa

**Keywords:** antibacterial, antifungal, biofilm, cytotoxicity, poultry pathogens

## Abstract

Antimicrobial resistant poultry pathogens are responsible for treatment failure and economic losses, and can also be a source of resistant zoonotic infections representing a risk to human health. In 2006 the European Union banned the use of antibiotics as growth promoters in farm animals and other regions are likely to follow suit. Alternative products and strategies are sought to help maintain animal gut health to reduce the prevalence of pathogens in the food chain. The minimum inhibitory concentration (MIC) of organic and aqueous leaf extracts of *Alchornea laxiflora, Ficus exasperata, Morinda lucida, Jatropha gossypiifolia, Ocimum gratissimum*, and *Acalypha wilkesiana* were tested against bacterial poultry pathogens including *Staphylococcus aureus, Enterococcus faecalis, Salmonella* spp., *Escherichia coli, Campylobacter* spp., and fungal species (*Aspergillus fumigatus, Aspergillus flavus*, and *Candida albicans)* using a 2-fold serial microdilution method. Activity of extracts against biofilms of the pathogens was done using a modified crystal violet staining *in vitro* assay. The safety of extracts was determined against Vero and Caco-2 cells using a tetrazolium-based *in vitro* assay. Acetone and cold water extracts of *M*. *lucida* had the best activity against three bacteria (MIC = 0.05–0.07 mg/ml) and two fungal (MIC = 0.03–0.15 mg/ml) organisms, respectively. The *E*. *coli* isolate and *A*. *flavus* were the most susceptible bacteria and fungi, respectively. Caco-2 cells generally displayed higher selectivity index (SI) values compared to Vero cells and average SI values against Vero and Caco-2 cells for both bacteria and fungi ranged from 0.01 to 4.48 and 0.005 to 16.41, respectively. All plant extracts had good anti-biofilm activity (>50%) against at least one organism. The disruption of established biofilm growth by the plant samples proved to be more difficult to achieve than efficacy against planktonic forms of bacteria. This study shows that some of the plant species are potential candidates as alternative feed additives in poultry production. In the future, a poultry feed trial to evaluate their *in vivo* efficacy as herbal feed additives will be conducted.

## Introduction

Poultry diseases caused by a number of pathogens compromise animal health and welfare and decrease production efficiencies, causing reduced profitability, and increased levels of antimicrobial use. The contamination of poultry food products with various zoonotic pathogens is also a concern to food safety and public health, while there is increased consumer awareness and demand for organic poultry products (1). Many pathogens such as *Salmonella* spp. and *Campylobacter* spp. form biofilms, which further exacerbate diseases in poultry and resistance to antimicrobials. Biofilms are complex biological structures consisting of many bacterial cells surrounded by layers of substances produced by them, forming a barrier hindering eradication of the organisms (2).

Antimicrobial agents are used extensively in poultry production and are usually administered in the feed or drinking water. The use of antimicrobials has undoubtedly contributed to the success of the poultry industry from large numbers of small-scale farmers to a smaller number of large-scale producers who operate at high efficiency (3, 4). However, the prolonged use of antibiotics at sub-therapeutic levels as feed additives in animal and poultry feeds is a major risk factor to the emergence of drug-resistant pathogens, with major negative impacts on human, animal and environmental health (5). Also, the reliance of the poultry industry on the use of antimicrobials to prevent as well as control infectious diseases highlights the risks to the financial sustainability of the sector from the continuing growth in farm bacterial reservoirs with resistance to antimicrobial treatments (6–8). Therefore, there is a need to uphold proper antimicrobial stewardship by limiting antimicrobial use in food animals especially as prophylaxis should be decreased or stopped to limit the impact of AMR (antimicrobial resistance) on human health (9). For example, in 2006, the European Union banned the use of antimicrobials for prophylaxis in livestock while the use of colistin for animal use was recently banned in China (10).

Generally, contamination of poultry-based foods by *Salmonella* organisms has placed poultry products at a higher risk compared to other foods. In 2012, several outbreaks of *Salmonella* were associated with poultry meat and products (www.cdc.gov/salmonella/outbreaks.html). In the course of slaughtering, *Salmonella* from the gastrointestinal tract of infected chickens can contaminate the carcasses and the processing line (11). *Salmonella* species have been reported to be globally widespread food-borne pathogens, of which outbreaks are commonly associated with the consumption of contaminated food such as eggs, poultry meat and pork. In the European Union, *Salmonella* is a major cause of food poisoning (12) and *Salmonella* was recognized as one of the major food-borne pathogens in the United States, causing an estimated 1.4 million cases of illness, with ~20,000 hospitalizations and more than 500 deaths annually (13).

In view of these challenges *vis a vis* the need to sustain profitability in livestock production without compromising public health safety, there is an urgent need for alternatives to prophylactic antimicrobial use. Phytogenic, or plant-based, additives are considered to be a promising alternative as non-antibiotic antimicrobials and potential feed additives to promote growth and increase production (14).

The use of medicinal plants as traditional medicines is well-known in rural areas of many developing countries (15, 16). Many plants have been used because of their antimicrobial traits derived from compounds which are chiefly synthesized during secondary metabolism of the plant (17). Several plant species were chosen for this study as a result of their reported antibacterial activity or traditional use to treat bacterial-related infections. *Alchornea laxiflora* (Benth) Pax and Hoffman (Euphorbiaceae) is commonly called Lowveld bead-string and is widely distributed from Nigeria to Ethiopia and down to Mpumalanga, South Africa. Akinpelu et al. (18) reported that the methanolic extract of this plant is a potent source of antibacterial and antifungal compounds. Reversal of sodium arsenate-induced liver toxicity by the hexane leaf extract in animal models was reported by Esosa et al. (19).

*Ficus exasperata* Vahl (Moraceae) is otherwise known as Sandpaper leaf (English), “Ewe Ipin or Eepin” (Yoruba-Western Nigeria), “Baure” (Hausa-Northern Nigeria, and “Asesa” (Igbo-Eastern Nigeria) (20). Fresh leaves are used in the local management of hypertension, rheumatism, arthritis, diarrhea, dysentery, intestinal pains and colic, epilepsy, oedema, gout, leprosy, bleeding, and wounds (21). The aqueous leaf extract of the plant had MIC values of 10, 20, and 10 mg/ml against *E*. *coli, S*. *aureus* and *E*. *faecalis*, respectively using macro broth dilution techniques (22). *Morinda lucida* L. (Rubiaceae) is a tropical West African rainforest species commonly known as Brimstone tree (23), and has been used in the traditional treatment of wound infections, diarrhea, malaria, diabetes, typhoid, abscesses, and chancre (24, 25).

*Jatropha gossypiifolia* L. (Euphorbiaceae) is widely distributed in countries of tropical, subtropical, and dry tropical weather as well as tropical semi-arid regions of Africa and the Americas. The leaves and bark were reported to have antimicrobial, anti-hypertensive, anti-inflammatory, analgesic, haemostatic and anti-diabetic properties (26, 27). *Ocimum gratissimum* L. (Labiateae) is found throughout the tropics and subtropics and its greatest variability occurs in tropical Africa and India (28). The extracts of leaves or whole plants of *O*. *gratissimum* are popular for the treatment of diarrhea and cold infusions of the leaves are used for the relief of stomach upset and hemorrhoids (29). The leaves have been reported to be rich in thymol which has antimicrobial properties (30). *Acalypha wilkesiana* (Euphorbiacae) is common in many countries, especially in the tropics of Africa, America and Asia. *A*. *wilkesiana* has antibacterial and antifungal properties (31, 32). The leaves of *A*. *wilkesiana* have various ethnomedicinal uses which include the treatment of malaria, and dermatological and gastrointestinal disorders (33).

Our previous research on some of the above-mentioned plant species revealed that *M*. *lucida, A*. *wilkesiana*, and *F*. *exasperata* leaves have appreciable amounts of macro- and microminerals, anions, sugars and organic acids, all of which are nutritional requirements of poultry. The plant species displayed appreciable levels of total phenolics and flavonoids which are most likely related to their antimicrobial potential (34). The antimicrobial and antioxidant properties of secondary metabolites, mostly phenolics and flavonoids, will enhance the potential of these plants as phytogenic feed additives (PFAs). This study will add knowledge to the application, safety and mode of action of phytogenics which comprise a relatively new class of feed additives in animal nutrition.

The objectives of this study were to determine the antimicrobial potential of extracts of the selected plant species against the planktonic forms and biofilms of some economically important infectious poultry disease agents. Furthermore, the *in vitro* cytotoxicity of the plant extracts was determined against two mammalian cell lines.

## Materials and Methods

### Plant Collection

Fresh leaves of *Alchornea laxiflora* (A.L), *Ficus exasperata* (F.E), *Morinda lucida* (M.L), *Jatropha gossypiifolia* (J.G), *Ocimum gratissimum* (O.G), and *Acalypha wilkesiana* (A.W) were collected from Ibadan Metropolis at Lagelu local Government Area of Oyo State, Nigeria in June 2017. The plants were identified by Mr Donatus Ozimede Esimekhinai. Voucher specimens were deposited after identification in the herbarium of the Department of Botany, University of Ibadan. Nigeria with numbers as follows: *A*. *laxiflora* (UIH-22625) *F*. *exasperata* (UIH-22626), *M*. *lucida* (UIH-2629), *J*. *gossypiifolia* (UIH-22627), *O*. *gratissimum* (UIH-22628), and *A*. *wilkesiana* (UIH-22793). The thoroughly cleaned and dried plants were ground into powder and kept in sealed containers in the dark until subsequent use.

### Plant Extraction and Preparation

Three grams of the powdered material of each plant were weighed into 50 ml centrifuge tubes and 30 ml of acetone, methanol, ethanol, cold distilled water, and hot distilled water were added to separate aliquots and macerated for 24 h. The mixtures were centrifuged at 300 × g for 10 min and then filtered through Whatman No. 1 filter paper. The resultant extracts were transferred into pre-weighed labeled glass vials and the procedure was repeated thrice to exhaustively extract plant material. Resultant extracts were placed under a stream of air to dry completely and stored in the dark at 4°C. The resultant extracts were reconstituted in their respective solvents to the desired concentrations for the study.

### Microbial Strains

Of the 18 microbial strains used in this study, eight were obtained from the American Type Culture Collection (ATCC), and 10 were clinical isolates. The ATCC strains used were *Staphylococcus aureus* (ATCC 29213), *Enterococcus faecalis* (ATCC 29212), *Salmonella enterica* subsp. *enterica* serovar Enteritidis (*S*. Enteritidis, ATCC 13076), *S*. Dublin (ATCC 15480), *S*. Typhimurium (ATCC 700720), *Escherichia coli* (ATCC 25922), *Campylobacter coli* (ATCC 43478), and *Campylobacter jejuni* (ATCC 33560). Clinical isolates obtained from the Department of Veterinary Tropical Diseases, University of Pretoria (UP) and included *Escherichia coli* (culture number B 3427/16), *S*. Gallinarum (B51/07), *S*. Idikan (B 1975/16), *S*. Bsilla (B 862/15), *S*. Choleraesuis (B 2209/17), *S*. Kottbus (B 297/16), *S*. Braenderup (AJ 42), *Aspergillus fumigatus* (isolated from a chicken with systemic mycosis,), and *Candida albicans* (isolated from a Gouldian finch). Avian crop candidiasis generally manifests itself as a localized infection of the mucous membranes, particularly crops (35). *Aspergillus flavus* was obtained from the Agricultural Research Council, South Africa, culture number PPRI 3954. Chickens have been reported to be exposed to feed contaminated with cyclopiazonic acid, a toxin produced by *A*. *flavus* which is a natural contaminant of corn (36) and peanuts (37).

### *In vitro* Antimicrobial Serial Microdilution Assay

The antibacterial and antifungal assays were carried out using microdilution methods as described by Eloff (38) and Masoko et al. (39). All the bacteria in this study, except the *Campylobacter* strains, were prepared by inoculating a single colony of each bacterial strain from an agar plate into sterilized MH (Mueller Hinton) broth and grown overnight in a shaking incubator. *Campylobacter* strains were inoculated into BHI (Brain Heart Infusion) broth and grown overnight under anaerobic conditions at 37°C. All fungal strains were inoculated into Sabouraud Dextrose (SD) broth and placed in a shaking incubator for 24 h for *C*. *albicans* and 72 h for *Aspergillus* species at 30°C. Each culture was adjusted to a McFarland standard No 1 (equivalent to 3 × 10^8^ cfu/ml). One hundred microliters of sterile water were added to each well of sterile 96-well microplates. Plant samples (100 μl) re-suspended to 10 mg/ml in sterile water for the water extracts, and acetone for the organic solvent extracts, were added to the first well of the microplates and then serially diluted along the ordinate. Gentamicin (Virbac) and amphotericin B (Sigma) were used as positive controls for the bacteria and fungi respectively while acetone and water served as negative controls. Subsequently, 100 μl of each of appropriately adjusted bacterial or fungal cultures were added to the wells of the microplates. The microplates were incubated at 37°C for bacteria and 30°C for fungi for 24 h. To each well of the incubated microplates, 40 μl of 0.2 mg/ml p-iodonitrotetrazolium (INT, Sigma) were added to bacteria and 50 μl to the fungal plates. The plates were further incubated at 37°C for 30 min before reading the MIC for bacteria while readings were taken after 24 and 48 h for fungi. The last well with clear inhibition of bacterial and fungal growth was recorded as the minimum inhibitory concentration (MIC).

### Chromatographic Analysis

Each plant extract (10 μL of a 10 mg/ml concentration) was loaded in a band of 1 cm on thin layer chromatography (TLC) Merck aluminum-backed plates (silica gel 60 F254) for chromatographic analysis. The TLC plates were later developed in three solvent systems of varying polarities (40), namely benzene: ethanol: ammonium hydroxide (90:10:1, BEA, non-polar basic), chloroform: ethyl acetate: formic acid (5:4:1, CEF, intermediate polarity, acidic), and ethyl acetate: methanol: water (40:5.4:5, EMW, polar, neutral). Separated chemical compounds were detected using acidified vanillin (0.1 g vanillin: 28 ml methanol: 1 ml sulphuric acid) as a spray. After spraying, the chromatograms were heated at 110°C in an incubator to allow for optimal color development.

### Bioautographic Analysis

Thin layer chromatography (TLC) plates were loaded with 10 μl of each plant extract at 10 mg/ml concentration. TLC plates were prepared and developed in the three different solvent systems described above, and dried overnight under a stream of air to remove residual solvent which might inhibit organism growth. The plates were sprayed with cultures of bacteria (*E*. *coli, S*. Enteritidis, *S*. *aureus*, and *C*. *jejuni*) and fungi (*A*. *fumigatus* and *C*. *albicans*) in fresh growth medium. The moist plates were incubated at 37°C at 100% relative humidity for 24 h. The plates were then sprayed with 2 mg/ml of INT (41) and further incubated for 1–2 h. The purple-red color was an indication of cell viability while clear zones against the purple background were indicative of antibacterial and antifungal activity of separated compounds.

### Anti-biofilm Assay

#### Inhibition of Bacterial Biofilm Formation

The inhibition of biofilm formation by acetone and aqueous (cold) extracts of the plants were assessed *via* the modified protocol by Sandasi et al. (42) and Mohsenipour and Hassanshahian (43). Two biofilm development stages were investigated, which were prevention of biofilm attachment (T0) and destruction of 24 h pre-formed biofilm (T24). The biofilm was allowed to preform for either 0 h (T0) or 24 h (T24) before the addition of samples (plant extracts) at a final concentration of 1 mg/ml. For the T0 study, 100 μl of the respective standardized bacterial culture (OD_590_ = 0.02 equivalent to 1.0 × 10^6^ CFU/ml) prepared in Tryptone Soy Broth (TSB) was inoculated into sterile flat bottomed 96-well microtitre plates followed by adding 100 μl of the plant samples and incubated for 24 h at 37°C without shaking. For T24, 100 of standardized cultures were pre-incubated for 24 h for biofilm growth, before addition of plant extracts. For both T0 and T24, appropriate control included: negative control (culture + TSB), positive control [culture + TSB + antibiotics (ciprofloxacin, gentamicin and tetracycline)], sample control (sample + TSB), antibiotic control (antibiotic + TSB), and media control (TSB only). After 24 h incubation, the modified crystal violet staining (CVS) assay (42) was performed to quantify the biofilm biomass.

#### Crystal Violet Staining (CVS) Assay

Following incubation as described above, the wells were carefully emptied and plates were washed at least three times with sterile distilled water to remove unattached or loosely attached cells. The plates were air-dried and then oven-dried at 60°C for 45 min. Then 150 μl of 96% methanol was added to the wells for 15–20 min to fix the adherent cells. The plates were emptied, and the adhered cells stained with 100 μl of 0.1% crystal violet solution for 20 min at room temperature. Excess stain was rinsed off by washing the plates at least five times with water. Thereafter, the biofilm biomass was evaluated semi-quantitatively by re-solubilizing the crystal violet stain bound to the adherent cells with 150 μl of 100% ethanol to destain the wells. The absorbance of the plates was read at 590 nm using a microplate reader (Epoch™ Microplate Spectrophotometer) after careful and gentle shaking. The mean absorbance (OD_590nm_) of the sample was determined and results expressed as percentage inhibition using the equation below (42).


$$Percentage\;(\%)\;inhibition=\frac{OD_{Negative\;control}\;-\;OD_{Sample}\;\times \;100}{OD_{Negative\;control}}$$

### *In vitro* Cytotoxicity Assay

The cytotoxicity test was carried out by screening the acetone and aqueous (cold water) extracts of the six plant species against monkey kidney cells (VERO) and human intestinal (Caco-2) cell lines using the tetrazolium-based colorimetric (MTT) assay described by Mosmann (44) and modified by McGaw et al. (45). These two extracts were chosen for their good and overall antimicrobial potentials alongside with the replication of the possible safety of mixtures of the plant powder with biological fluids (chicken gut fluids). Both cell lines were maintained in Minimal Essential Medium (MEM) supplemented with 0.1% gentamicin (Virbac) and 5% (for VERO) or 10 % (for Caco-2) fetal calf serum (Highveld Biological) at 37°C in a 5% CO_2_ incubator till confluency. Cells of a sub-confluent culture were harvested and centrifuged at 700 × g for 7 min and resuspended in MEM to 5 × 10^4^ cells/ml. Cell suspension (100 μl) was pipetted into each well of columns 2 to 11 of a tissue culture grade sterile 96 well microtitre plate and only MEM (200 μl) was pipetted in columns 1 and 12 to minimize the “edge effect” and maintain humidity. The plates were incubated for 24 h at 37°C in a 5% CO_2_ incubator to allow for cell attachment e. Different concentrations of the extracts prepared in the complete media were added to the plates in quadruplicate with 2 repeats (*n* = 8). The microtitre plates were then incubated at 37°C in a 5% CO_2_ incubator for 48 h. Doxorubicin (Pfizer Laboratories) and acetone served as the positive control and negative controls, respectively. The contents of the cells were discarded and washed with phosphate buffered saline and replaced with 200 μl of fresh MEM. Then, 30 μl MTT (Sigma, stock solution of 5 mg/ml in PBS) was added to each well and the plates were incubated for a further 4 h at 37°C. The medium was aspirated and MTT formazan crystals were dissolved with 50 μl dimethyl sulphoxide (DMSO). The plates were shaken gently on an orbital shaker to allow the formazan to dissolve. The amount of MTT reduction was measured immediately by detecting absorbance in a microplate reader at a wavelength of 570 nm. The half maximal lethal concentration (LC_50_) value was calculated. Selectivity index (SI) values for antimicrobial activity were calculated using the formula SI = LC_50_/MIC.

### Total Activity (TA)

The total antibacterial activity of extracts was calculated by dividing the quantity extracted from 1 g of plant material with the MIC values obtained against bacteria or fungi in mg/ml (46). It indicates the amount in ml to which an extract from 1 g of plant material can be diluted and still inhibit the growth of the test organism.

## Results

### Plant Extract Yields

**Figure 1:**
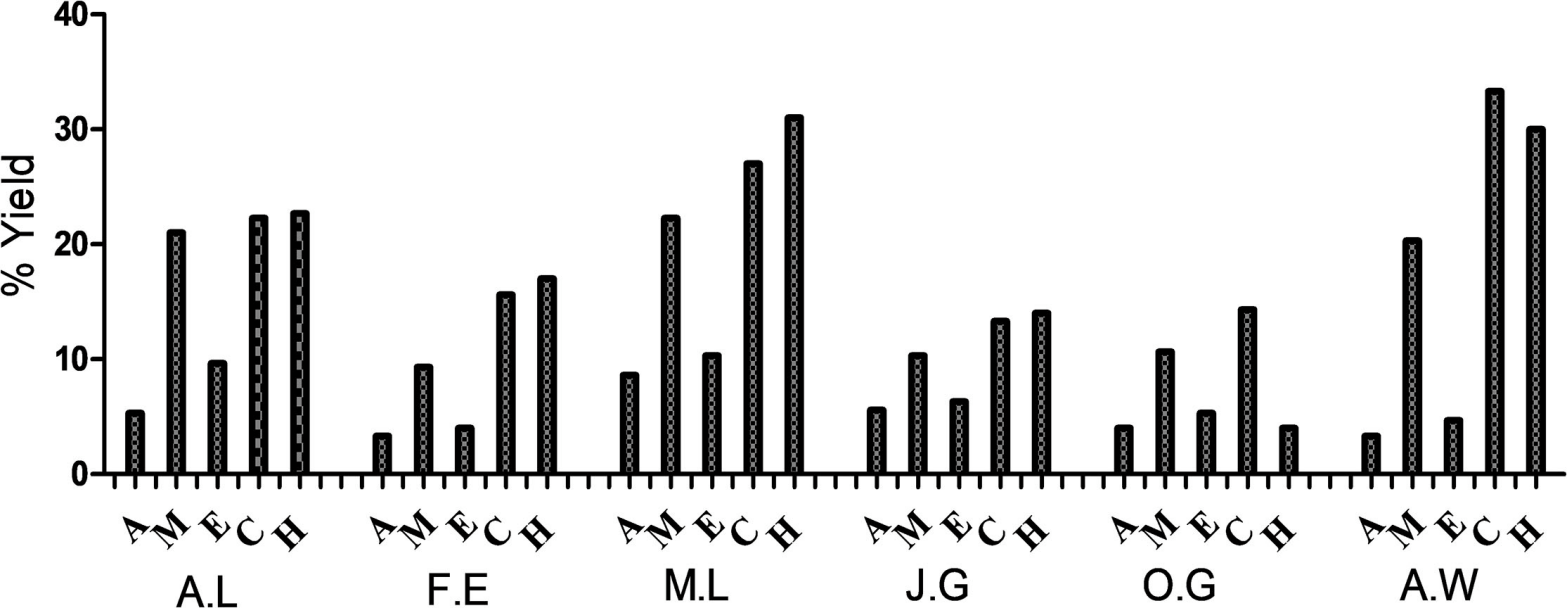
Percentage yields of the dried plant material. A.L, *A. laxiflora*; F.E, *F. exasperata;* M.L, *M. lucida*; J.G, *J. gossypiifolia*; O.G, *O. gratissimum*; A.W, *A. wilkesiana*; A, Acetone; M, Methanol; E, Ethanol; C, Cold water; H, Hot water.

The percentage yields of the dried plant material extracted by each of the solvents (acetone, methanol, ethanol, cold water, and hot water) used in this study are represented in Figure 1. Cold water extracted the highest quantity of plant material. The highest quantity was extracted from *A*. *wilkesiana* (33%) followed by the hot water extracts of *M*. *lucida* (31%) and *A*. *wilkesiana* (30%), respectively. The yield of the acetone extracts of *F*. *exasperata* and *A*. *wilkesiana* were lowest with 3.33%, respectively.

### Antibacterial Activity

Generally, among the extractants used for this study, the extracts of the tested plants prepared using acetone had the best average antimicrobial activity against the tested pathogens (Tables 1, 2).

**Table 1:** Antibacterial activity of the extracts of six selected plants against both Gram-positive and Gram-negative bacteria affecting chickens.

**Organisms**	**MIC (mg/ml)**																
	***Alchornea laxiflora***					***Ficus exasperate***					***Morinda lucida***					**Controls**	
	**A**	**M**	**E**	**C.W**	**HW**	**A**	**M**	**E**	**C.W**	**HW**	**A**	**M**	**E**	**C.W**	**HW**	**Ac**	**Gent**
*Staphylococcus aureus*	0.46*	0.62	0.38*	2.50	2.50	0.31*	1.87	2.50	0.31*	0.70	0.15*	2.50	2.50	2.50	2.50	>2.50	<0.01
*Enterococcus faecalis*	0.38*	0.31*	0.38*	1.40	1.25	0.19*	1.87	2.50	>2.50	1.87	0.23*	2.50	1.25	1.32	1.25	>2.50	<0.01
*Salmonella* Enteritidis	0.38*	0.62	0.31*	1.87	2.50	0.23*	1.25	0.31*	0.72	1.56	0.23*	1.25	1.25	2.50	0.62	>2.50	<0.01
*Salmonella* Gallinarum	0.15*	0.51*	0.31*	1.25	2.08	0.22*	0.62	0.51*	1.25	2.50	**0.07**	0.51*	0.83	2.50	2.50	>2.50	0.0003
*Escherichia coli* (ATCC)	0.31*	0.62	0.38*	2.50	1.25	0.78	0.78	0.62	1.25	1.87	0.46*	0.62	1.25	2.50	1.25	>2.50	<0.01
*Escherichia coli* (isolate)	**0.05**	0.51*	0.34*	**0.03**	>2.50	**0.07**	0.15*	1.04	1.25	0.15*	**0.07**	1.66	0.51*	**0.03**	0.15*	>2.50	0.001
*Campylobacter coli*	0.11*	0.62	0.34*	2.50	2.50	**0.05**	2.50	0.41*	2.50	2.50	**0.05**	0.83	0.41*	0.46*	2.50	>2.50	0.62
*Campylobacter jejuni*	0.22*	1.66	0.23*	2.50	>2.50	0.30*	1.25	0.83	2.50	1.87	0.18*	1.04	0.83	2.50	>2.50	>2.50	0.001
Average	0.26*	0.68	0.33*	1.82	2.13	0.27*	1.29	1.09	2.50	1.62	0.18*	1.36	1.10	1.79	1.65		
	***Jatropha gossypiifolia***					***Ocimum gratissimum***					***Acalypha wilkesiana***					**Controls**	
*Staphylococcus aureus*	0.31*	0.62	0.62	0.62	0.31*	1.25	2.50	2.50	2.50	2.50	0.62	0.62	0.15*	**0.07**	2.50	>2.50	<0.01
*Enterococcus faecalis*	0.23*	0.62	0.93	1.25	1.25	1.25	>2.50	2.50	1.32	1.25	**0.03**	**0.05**	**0.03**	1.25	1.25	>2.50	<0.01
*Salmonella* Enteritidis	0.15*	0.62	0.62	0.62	0.62	0.62	1.25	>2.50	1.87	1.25	0.31*	**0.07**	**0.07**	0.66	0.66	>2.50	<0.01
*Salmonella* Gallinarum	0.15*	**0.09**	**0.09**	1.25	2.08	0.31*	0.83	0.31*	1.25	2.08	0.15*	**0.09**	0.31*	1.25	**0.09**	>2.50	0.003
*Escherichia coli (*ATCC*)*	0.46*	1.25	0.93	1.25	1.25	0.62	2.50	2.50	1.56	0.62	0.62	0.93	0.62	2.50	**0.07**	>2.50	<0.01
*Escherichia coli* (isolate*)*	0.11*	0.83	0.41*	0.62	1.25	0.15*	1.66	1.04	**0.07**	1.25	0.46*	0.46*	0.62	1.25	**0.03**	>2.50	0.001
*Campylobacter coli*	**0.05**	**0.09**	0.20	0.62	1.66	0.31*	0.83	0.41*	1.25	0.83	0.46*	0.46*	0.83	2.50	0.62	>2.50	0.62
*Campylobacter jejuni*	**0.04**	0.20*	0.20	1.25	>2.50	0.31*	0.41*	0.31*	2.50	>2.50	**0.03**	0.31*	0.62	2.50	1.25	>2.50	0.001
Average	0.19*	0.54*	0.50*	0.94	1.36	0.60*	1.56	1.51	1.54	1.53	0.34*	0.37*	0.41*	1.50	0.80		

*A, Acetone; M, Methanol; E, Ethanol; CW, Cold water; HW, Hot water; Ac, Acetone; Gent, Gentamicin*.

*Bold values: MIC < 0.1 mg/ml (significantly active)*.

* *Moderate activity*.

**Table 2:** Antifungal activity of the extracts of six selected plants against chicken fungi.

**Organisms**	**Time (hr)**	**MIC (mg/ml)**																
		***Alchornea laxiflora***					***Ficus exasperata***					***Morinda lucida***					**Controls**	
		**A**	**M**	**E**	**C.W**	**HW**	**A**	**M**	**E**	**C.W**	**HW**	**A**	**M**	**E**	**C.W**	**HW**	**Ac**	**Amp**
*Aspergillus fumigatus* (isolate)	48	1.25*	1.25*	1.56	**0.31**	>2.50	**0.46**	0.93*	1.25*	0.62*	0.62*	1.56	1.25*	1.25*	**0.15**	2.50	>2.5	0.15
	72	1.25*	1.25*	1.56	**0.31**	1.25*	>2.50	1.56	1.25*	0.62*	0.62*	1.56	1.25*	1.25*	**0.15**	2.50	>2.5	0.62
*Aspergillus flavus*	48	0.62*	>2.50	2.50	>2.50	>2.50	**0.31**	>2.50	2.50	**0.15**	2.50	**0.15**	2.50	2.50	**0.31**	2.50	>2.5	0.62
	72	**0.46**	>2.50	2.50	>2.50	>2.50	**0.23**	2.50	0.51*	**0.15**	**0.38**	**0.19**	>2.50	>2.50	**0.03**	0.83*	>2.5	0.62
*Candida albicans* (isolate)	48	1.25*	2.50	1.87	>2.50	>2.50	>2.50	0.62*	0.62*	0.62*	0.93*	**0.46**	1.25*	2.50	>2.50	>2.50	>2.5	0.15
	72	0.93*	2.50	1.87	>2.50	>2.50	>2.50	0.93*	0.62*	0.62*	0.78*	**0.46**	2.50	2.50	>2.50	1.25*	>2.5	0.62
Average		1.00*	2.08	1.98	1.77	2.29	1.42*	1.51*	1.13*	**0.46**	0.97*	0.73*	1.88	2.08	0.94*	2.01		
		***Jatropha gossypiifolia***					***Ocimum gratissimum***					***Acalypha wilkesiana***					**Controls**	
*Aspergillus fumigatus* (isolate)	48	**0.31**	0.62*	0.62*	**0.31**	>2.50	2.50	1.25*	2.50	1.40*	>2.50	**0.07**	2.50	**0.15**	2.50	>2.50	>2.5	0.15
	72	**0.31**	2.50	1.25*	>2.50	>2.50	2.50	1.25*	2.50	0.62*	>2.50	0.62*	2.50	0.62*	2.50	1.25*	>2.5	0.62
*Aspergillus flavus*	48	0.62*	0.83*	0.62*	**0.15**	2.50	**0.03**	1.66	2.50	**0.31**	>2.50	1.25*	>2.50	**0.15**	>2.50	0.62*	>2.5	0.62
	72	0.93*	0.83*	1.25*	**0.15**	0.51	**0.15**	1.66	1.66	**0.31**	>2.50	0.62*	>2.50	0.62*	>2.50	**0.46**	>2.5	0.62
*Candida albicans* (isolate)	48	0.93*	1.25*	1.25*	2.50	2.50	1.25*	2.50	>2.50	1.56	0.62*	**0.15**	2.50	**0.15**	2.50	>2.50	>2.5	0.15
	72	1.25*	1.25*	1.25*	1.25*	2.50	1.25*	2.50	>2.50	1.87	0.62*	**0.15**	2.50	1.87	2.50	1.25*	>2.5	0.62
Average		0.73*	1.21*	1.04*	1.14*	2.16	1.28*	1.80	2.36	1.01*	1.87	**0.48**	2.50	0.60*	2.50	1.43*		

*A, Acetone; M, Methanol; E, Ethanol; CW, Cold water; HW, Hot water; Ac, Acetone; Amp, Amphotericin B*.

*MIC ≤ 0.5 mg/ml—Significantly active (Bold)*.

* *Moderate activity*.

The acetone and ethanol extracts of *Alchornea laxiflora* (A.L) had the lowest average MIC values for all tested organisms compared to other extracts of this plant with MIC values of 0.26 and 0.33 mg/ml, respectively (Table 1). The hot and cold water extracts of A.L had poor inhibitory effect on the growth of the bacterial strains tested as their MIC values were all >0.625 mg/ml. However, the cold water extracts of this plant had the best activity against *Escherichia coli* (isolate) with MIC of 0.03 mg/ml.

The acetone and ethanol extracts of *Ficus exasperata* had the lowest average MIC value against all tested organisms compared to other extracts of this plant with MIC values of 0.27 and 1.09 mg/ml, respectively (Table 1). The most sensitive organisms to the plant extracts were *C*. *coli* and *E*. *coli* (isolate) while *E*. *faecalis, E*. *coli* (ATCC), and *C*. *jejuni* were relatively resistant.

The acetone and ethanol extracts of *Morinda lucida* had the lowest average MIC value for all tested organisms compared to other extracts of this plant with average MIC values of 0.18 and 1.10 mg/ml, respectively (Table 1). The acetone extract displayed high activity against *Salmonella* Gallinarum, *E*. *coli* (isolate) and *C*. *coli* with MIC values of 0.07, 0.07, and 0.05 mg/ml, respectively. Similarly, the cold water extract had the best activity against *E*. *coli* (isolate) with MIC value of 0.03 mg/ml. The acetone extract had significant activity against *C*. *jejuni* with MIC values of 0.04 mg /ml. Both ethanol and methanol extracts had significant activity against *S*. Gallinarum with MIC value of 0.09 and 0.09 mg/ml, respectively. In addition, the methanol extract had high activity against *C*. *coli* with MIC value of 0.09 mg/ml (Table 1).

The acetone extract of *Ocimum gratissimum* had the best average MIC values for all tested organisms with MIC values of 0.60 mg/ml. The cold water extract had the best activity against *E*. *coli* (isolate) while the extracts had poor activity against *S*. *aureus, E*. *faecalis, S*. *Enteritiditis*, and *E*. *coli* (ATCC).

The acetone, methanol and ethanol extracts of *Acalypha wilkesiana* had the lowest average MIC values against all tested organisms compared to other extracts of this plant with MIC values of 0.34, 0.37, and 0.41 mg/ml, respectively (Table 1). The acetone, methanol and ethanol extracts displayed very good activities against *E*. *faecalis* with MIC values of 0.03, 0.05, and 0.03 mg/ml, respectively.

The average total antibacterial activity (TAA) values of the selected plants ranged from 61 to 5 989 ml/g against all tested bacteria (Table 3). The highest TAA of 40 666 ml/g (Table 3) was produced by the methanol extract of *A*. *wilkesiana* against *E*. *faecalis* which indicates that 1 g of *A*. *wilkesiana* can be diluted in 40 666 ml of the solvent used and still inhibit the growth of the organism.

**Table 3:** Percentage yields and total antibacterial activity (TAA) of the extracts of six selected plants against bacteria.

**Organisms**	**Total activity (ml/g)**														
	***Alchornea laxiflora***					***Ficus exasperata***					***Morinda lucida***				
	**A**	**M**	**E**	**C.W**	**HW**	**A**	**M**	**E**	**C.W**	**HW**	**A**	**M**	**E**	**C.W**	**HW**
*Staphylococcus aureus*	115.93	338.70	254.38	89.33	90.67	107.50	49.91	16.00	505.38	242.86	577.78	89.33	41.33	108.00	124
*Enterococcus faecalis*	140.35	677.41	254.39	159.52	181.33	175.44	49.91	16.00	62.67	90.91	376.81	89.33	82.67	204.54	248
*Salmonella* Enteritidis	140.34	338.71	311.84	119.42	90.66	144.91	74.66	129.03	217.60	109	376.78	178.66	82.66	108.00	500
*Salmonella* Gallinarum	355.53	411.76	311.84	178.66	108.97	151.5	150.53	78.43	125.34	68	1,238	437.90	124.49	108	124
*Escherichia coli* (ATCC)	172.03	338.71	254.39	89.33	181.33	42.73	119.65	64.52	125.34	90.91	188.39	360.20	82.66	108	248
*Escherichia coli* (Isolate)	1,066.6	411.76	284.32	7,444.3	90.66	476.14	622.2	38.46	125.34	1,133.33	1,238	134.54	202.60	9,000	2,066.67
*Campylobacter coli*	484.81	338.71	284.32	89.33	90.66	666.6	37.33	97.56	62.67	68	1,733.2	269.07	252.02	586.96	124
*Campylobacter jejuni*	242.41	126.51	420.30	89.33	90.66	111.1	74.66	48.19	62.67	90.91	481.44	214.74	124.49	108	124
% Yield	5.33	21.00	9.66	22.30	22.67	3.33	9.33	4.00	15.60	17.00	8.60	22.30	10.30	27.00	31.00
Average	339.75	372.78	296.97	1,032.40	115.62	234.49	147.36	61.02	160.88	236.74	776.3	221.72	124.12	1,291.44	444.83
	***Jatropha gossypiifolia***					***Ocimum gratissimum***					***Acalypha wilkesiana***				
*Staphylococcus aureus*	182.79	268.82	102.15	215.05	451.61	32	42.67	21.33	57.33	66.66	53.76	327.96	311.11	4,761.90	120
*Enterococcus faecalis*	246.38	166.67	68.10	106.67	112	32.00	42.67	21.33	108.59	133.33	1,111.11	40,666.67	1,555.56	266.67	240
*Salmonella* Enteritidis	377.80	166.66	102.15	215.05	225.81	32	85.34	21.33	76.65	133.33	107.52	2,904.71	666.71	505.05	454.54
*Salmonella* Gallinarum	377.8	1,148.11	703.67	106.67	67.30	129.03	128.51	172.03	114.66	80.13	222.2	2,259.22	150.55	266.66	3,333.33
*Escherichia coli (ATCC)*	123.20	82.66	68.10	106.67	112	64.52	42.67	21.33	91.88	268.81	53.75	218.63	75.27	133.33	4285.71
*Escherichia coli (Isolate)*	515.18	124.49	154.46	215.05	112	266.67	64.25	51.28	2,047.57	133.33	72.46	442.02	75.27	266.66	10,000
*Campylobacter coli*	1,133.4	1,148.11	316.65	215.05	84.34	129.03	128.51	130.07	114.66	200.80	72.46	442.02	56.23	133.33	483.87
*Campylobacter jejuni*	1,416.75	516.65	316.65	106.66	56	129.03	260.15	172.03	57.33	66.66	1,111	655.90	75.27	133.33	240
% Yield	5.60	10.33	6.33	13.33	14.00	4.00	10.66	5.33	14.33	4.00	3.33	20.33	4.67	33.33	30.00
Average	546.66	452.77	228.99	160.86	152.63	101.79	99.35	76.35	333.58	135.38	350.53	5,989.64	37,075	808.37	2,394.68

*A, Acetone; M, Methanol; E, Ethanol; CW, Cold water; HW, Hot water*.

### Antifungal Activity

The acetone and cold water extracts of *A*. *laxiflora, F*. *exasperata, M*. *lucida, J*. *gossypiifolia, O*. *gratissimum*, and *A*. *wilkesiana* had high antifungal activity with MIC values ranging from 0.03 to 0.48 mg/ml against one or more of the tested microorganisms (Table 2). The acetone and cold water extracts of *A*. *laxiflora* (A.L) had good overall antifungal activity for all tested organisms with MIC of 1.00 and 1.77 mg/ml, respectively. The cold water extract of A.L had the highest antifungal activity against *Aspergillus fumigatus* (isolate) with MIC value of 0.31 mg/ml while the acetone extract of the same plant had MIC value of 0.46 mg/ml against *Aspergillus flavus*. *Candida albicans* (isolate) was resistant to the plant extracts of A.L.

The cold water extract of *F*. *exasperata* had the lowest average MIC value for all the tested organisms with MIC value of 0.46 mg/ml. The acetone extract had moderate antifungal activity against *A*. *fumigatus* and *A*. *flavus* with MIC values of 0.46 and 0.31 mg/ml, respectively while the cold water extract had good antifungal activity against *A*. *flavus*.

The best overall average antifungal activity against all tested organisms was displayed by acetone and cold water extracts of *M*. *lucida* with MIC values of 0.73 and 0.94 mg/ml, respectively. Acetone and cold water extracts of *O*. *gratissimum* were the least active against all tested organisms with MIC values of 1.28 and 1.01 mg/ml, respectively (Table 2).

The acetone extract of *A*. *wilkesiana* had the lowest average MIC against all tested organisms with MIC of 0.48 mg/ml. This extract was active against both *A*. *fumigatus* and *C*. *albicans* with MIC values of 0.07 and 0.15 mg/ml, respectively. The acetone and cold water extracts of all the plants had antifungal activity against at least one or more of the tested fungi (Table 2).

Generally, the average total antifungal activity (TAA) values against tested fungi ranged from 18 to 2 281 ml/g (Table 4). The cold water extract of *M*. *lucida* had highest values of average total TAA against *A*. *flavus* over an incubation period of 72 h. The higher TAA indicates the levels of usefulness and economic values of the selected plant species.

**Table 4:** Percentage yields and total antifungal activity (TAA) of the extracts of six selected plants against chicken fungi.

**Organisms**	**Time (h)**	**Total activity (ml/g)**														
		***Alchornea laxiflora***					***Ficus exasperata***					***Morinda lucida***				
		**A**	**M**	**E**	**C.W**	**HW**	**A**	**M**	**E**	**C.W**	**HW**	**A**	**M**	**E**	**C.W**	**HW**
*Aspergillus fumigatus* (isolate)	48	42.66	168	61.97	720.42	90.66	72.46	100.35	32	252.69	274.19	55.55	178.66	82.66	1,800	124
	72	42.66	168	61.97	720.42	181.33	13.33	59.83	32	252.69	274.19	55.55	178.66	82.66	1,800	124
*Aspergillus flavus*	48	86.02	84	38.67	89.33	90.66	107.52	37.33	16	1,044.47	68	577.73	89.33	41.33	870.97	124
	72	115.93	84	38.67	89.33	90.66	144.91	37.33	78.43	1,044.47	447.37	456.11	89.3	41.33	9,000	373.50
*Candida albicans* (Isolate)	48	42.66	84	51.70	89.33	90.66	13.33	150.53	64.52	252.69	182.80	188.39	178.67	41.33	108	124
	72	57.34	84	51.70	89.33	90.66	13.33	100.35	64.52	252.69	217.94	188.39	89.33	41.33	108	248
% Yield		5.33	21.00	9.66	22.30	22.67	3.33	9.33	4.00	15.60	17.00	8.60	22.30	10.30	27.00	31.00
Average		64.54	112	50.78	299.69	105.77	60.81	80.95	47.91	516.62	244.08	253.62	133.99	55.11	2,281.16	186.25
		***Jatropha gossypiifolia***					***Ocimum gratissimum***					***Acalypha wilkesiana***				
*Aspergillus fumigatus* (isolate)	48	182.81	166.66	102.15	430.10	56	16	85.33	21.33	102.38	66.66	476.14	81.33	311.13	18.67	120
	72	182.81	41.33	50.66	53.33	56	16	85.33	21.33	231.18	66.66	53.76	81.33	75.27	18.67	240
*Aspergillus flavus*	48	91.40	124.49	102.15	888.87	56	1,333.33	64.25	21.33	462.35	66.66	26.66	81.33	311.13	18.67	483.87
	72	60.94	124.49	50.66	888.87	274.51	266.67	64.25	32.13	462.35	66.66	53.76	81.33	75.27	18.67	652.17
*Candida albicans* (isolate)	48	60.94	82.66	50.66	53.33	56	32	42.66	21.33	91.88	268.80	222.2	81.33	311.13	18.67	120
	72	45.34	82.66	50.66	106.66	56	32	42.66	21.33	76.65	268.80	222.2	81.33	24.96	18.67	240
% Yield		5.60	10.33	6.33	13.33	14.00	4.00	10.66	5.33	14.33	4.00	3.33	20.33	4.67	33.30	30.00
Average		104.37	103.72	67.82	403.53	92.42	282.67	64.08	23.13	237.80	134.04	175.79	81.33	184.82	18.67	309.34

*A, Acetone; M, Methanol; E, Ethanol; CW, Cold water; HW, Hot water*.

### Chromatographic Analysis

The Supplementary Figure 1 revealed the chemical fingerprint of the extracts. The plates showed different compounds separated with the different solvent systems from non-polar (BEA), to intermediately polar (CEF), and polar (EMW) solvent systems. This plate gives a qualitative overview of the compounds present in the extracts using vanillin as a spray reagent.

### Bioautographic Analysis

All three solvent systems separated the active bands against the tested microorganisms except against the fungi where CEF and EMW separated better than BEA. Using three solvent systems, 77 active bands were seen for the test organisms in the chromatographs of the different plant extracts. The CEF system separated 33 (42.86%) of the active bands followed by EMW with 29 (37.66%) and BEA with 15 (19.48%) which implies that most of the compounds were more polar in nature. *J*. *gossypiifolia* had the highest number of clear zones of inhibition representing active compounds against *E*. *coli* (Supplementary Figure 2, *S*. Enteritidis (Supplementary Figure 3), and *S*. *aureus* (Supplementary Figure 4). *M*. *lucida* had the highest number of active bands against *C*. *jejuni* (Supplementary Figure 5). However, no clear zone of inhibition was observed against *A*. *fumigatus* and *C*. *albicans* (Supplementary Figures 6, 7).

### Anti-biofilm Activity

The results of anti-biofilm (ABF) potential of the plant extracts against selected chicken pathogens are presented in Table 5. Extracts or fractions resulting in inhibition above 50% were considered to have good ABF activity (++) while those with inhibition between 0 and 50% indicated poor ABF activity (+), and the values <0 (-) were regarded as no inhibition, or enhancement of biofilm development and growth (47). All the tested extracts, except for the aqueous extract of *M*. *lucida* had good inhibitory activity against the planktonic cells of *E*. *coli* (Table 5). Acetone extracts of *F*. *exasperata* and *O*. *gratissimum* had good ABF potential (>50% inhibition) against *E*. *coli*. Acetone extracts of *M*. *lucida, A*. *laxiflora, F*. *exasperata, O*. *gratissimum*, and *A*. *wilkesiana*, as well as the aqueous extract of *O*. *gratissimum*, had good inhibitory activity (>50% inhibition) against the planktonic cells of *C*. *coli* (Table 5). All the extracts enhanced the formation of biofilm by *C*. *coli* and *Salmonella* Gallinarum (Table 5). Aqueous extracts of *M*. *lucida, A*. *laxiflora, F*. *exasperata, O*. *gratissimum, A*. *wilkesiana, J*. *gossypiifolia*, and the acetone extract of *J*. *gossypiifolia* had good ABF activity (>50% inhibition).

**Table 5:** Anti-biofilm activity of acetone and aqueous extracts of plant extracts against selected poultry pathogens.

**Plants**	**Solvents**	**% inhibition** **(*****E***. ***coli*****)**		**% inhibition** **(C**. ***coli*****)**		**% inhibition** **(*****C***. ***jejuni*****)**		**% inhibition** **(*****S*****. Gallinarum)**	
		**T_**0**_**	**T_**24**_**	**T_**0**_**	**T_**24**_**	**T_**0**_**	**T_**24**_**	**T_**0**_**	**T_**24**_**
*M*. *lucida*	Acetone	++	-	++	-	+	-	++	-
	Aqueous	-	-	-	-	-	++	-	-
*A*. *laxiflora*	Acetone	++	-	++	-	++	-	++	-
	Aqueous	++	-	-	-	-	++	-	-
*F*. *exasperata*	Acetone	++	++	++	-	++	-	++	-
	Aqueous	++	-	-	-	-	++	-	-
*O*. *gratissimum*	Acetone	++	++	++	-	++	-	++	-
	Aqueous	++	-	++	-	-	++	++	-
*J*. *gossypiifolia*	Acetone	++	+	-	-	++	++	-	-
	Aqueous	++	-	-	-	-	++	+	-
*A*. *wilkesiana*	Acetone	++	-	++	-	-	++	++	-
Gentamicin		-	++	++	-	-	++	++	1.04
Ciprofloxacin		++	++	-	-	-	-	++	-
Tetracycline		++	++	++	-	++	-	++	125.58

*Good (++) ABF activity (> 50% inhibition); Poor (+) ABF activity (more than 0–50% inhibition); No (-) ABF activity (0 % or less); Aqueous: Cold water*.

The results of the percentage inhibition of acetone and aqueous extracts against biofilm formation of eight *Salmonella* serovars is presented in Table 6. Acetone extracts of *M*. *lucida* had good inhibitory activity (>50% inhibition) against planktonic cells of all the organisms except *S*. Typhimurium. Acetone extracts of *M*. *lucida* showed good ABF activity (>50% inhibition) against *Salmonella* Cholerasuis, *S*. Idikan, *S*. Kottbus, and *S*. Enteritidis. Similarly, aqueous extracts of *M*. *lucida* also exhibited good ABF activity (>50% inhibition) against *S*. Dublin, *S*. Idikan, *S*. Kottbus, and *S*. Typhimurium. Also, the acetone extract of *M*. *lucida* had good ABF activity (> 50 % inhibition) (Table 6). The above results showed that the inhibition of biofilm formation by the extracts at T_0_ was higher compared to inhibition at T_24_, since the cells are still floating at T_0_ and not properly attached compared to those at T_24_, which reflects a more established biofilm.

**Table 6:** The percentage inhibition of acetone, aqueous extracts of *M*. *lucida* against biofilm formation of eight *Salmonella* serovars.

**Organisms**		**% inhibition**						
	**T**_**0**_				**T**_**24**_			
	***Morinda lucida***		Positive controls*		***Morinda lucida***		Positive controls*	
	**Acetone**	**Aqueous**	**Gent**	**Cipro**	**Acetone**	**Aqueous**	**Gent**	**Cipro**
*S*. Gallinarum	++	-	++	++	-	-	+	-
*S*. Dublin	++	-	-	++	-	++	-	-
*S*. Choleraesuis	++	-	-	++	++	-	-	++
*S*. Braenderup	++	-	+	+	-	-	++	-
*S*. Idikan	++	+	+	-	++	++	++	-
*S*. Kottbus	++	-	-	+	++	++	++	-
*S*. Typhimurium	-	-	-	-	-	++	-	-
*S*. Enteritidis	++	-	-	+	++	-	-	+

* *Positive controls: Gent, gentamicin; Cipro, ciprofloxacin. Good (++) ABF activity (> 50% inhibition); Poor (+) ABF activity (more than 0–50% inhibition); No (-) ABF activity (0 % or less)*.

### Cytotoxicity and Selectivity Index (SI)

The cold water extract of *A*. *laxiflora* had the highest LC_50_ value (lowest toxicity) of 0.709 mg/ml followed by the cold water extract of *Morinda lucida* with LC_50_ of 0.333 mg/ml (Table 7). The acetone extract of *J*. *gossypiifolia* was the most toxic (LC_50_ = 0.023 mg/ml) against Vero cells. The cold water extract of *Morinda lucida* had the highest LC_50_ value (lowest toxicity) of 0.580 mg/ml followed by the cold water extract of *F*. *exasperata* with LC_50_ value of 0.575 mg/ml while the acetone extract of *J*. *gossypiifolia* was the most toxic with LC_50_ value of 0.003 mg/ml against Caco-2 cells (Table 7). Both acetone and cold water extracts of *A*. *laxiflora, F*. *exasperata, Morinda lucida, J*. *gossypiifolia, O*. *grattisium*, and *A*. *wilkesiana* were not toxic to Caco-2 cells except for the acetone extract of *J*. *gossypiifolia*.

**Table 7:** Cytotoxicity (LC_50_ in mg/ml) and selectivity index (SI) of plant extracts and selectivity index against Vero kidney and Caco-2 cells.

**Plant species**	**Extracts**	**Selectivity index**																			
		**Vero cells**										**Caco-2 cells**									
		**LC_**50**_**	**Sa**	**Ef**	**Se**	**Sg**	**Ec (T)**	**Ec (iso)**	**Cc**	**Cj**	**Average**	**LC_**50**_**	**Sa**	**Ef**	**Se**	**Sg**	**Ec (T)**	**Ec (iso)**	**Cc**	**Cj**	**Average**
*A*. *laxiflora*	Acetone	0.026	0.06	0.07	0.07	0.17	0.08	0.52	0.24	0.12	0.17	0.026	0.06	0.07	0.07	0.17	0.08	0.52	0.24	0.12	0.17
	Cold water	0.709	0.28	0.51	0.38	0.57	0.28	**26.33**	0.28	0.28	**3.61**	0.417	0.17	0.30	0.22	0.33	0.17	**13.90**	0.17	0.17	**1.93**
*F*. *exasperata*	Acetone	0.099	0.32	0.52	0.43	0.45	0.13	**1.41**	**1.98**	0.33	0.70	0.561	**1.81**	**2.95**	**2.44**	**2.55**	0.72	**8.01**	**11.22**	**1.87**	**3.95**
	Cold water	0.164	0.53	0.07	0.23	0.13	0.13	0.13	0.07	0.07	0.17	0.575	**1.85**	0.23	0.80	0.46	0.46	0.46	0.23	0.23	0.59
*M*. *lucida*	Acetone	0.033	0.22	0.14	0.01	0.47	0.07	0.47	0.66	0.18	0.28	0.041	0.27	0.18	0.18	0.59	0.09	0.59	0.82	0.23	0.37
	Cold water	0.333	0.13	0.25	0.13	0.13	0.13	**11.10**	0.72	0.13	**1.59**	0.580	0.23	0.44	0.23	0.23	0.23	**19.33**	**1.26**	0.23	**2.77**
*J*. *gossypiifolia*	Acetone	0.023	0.07	0.08	0.15	0.15	0.05	0.21	0.46	0.58	0.22	0.003	0.01	0.01	0.02	0.02	0.01	0.03	0.06	0.08	0.03
	Cold water	0.144	0.23	0.12	0.23	0.12	0.12	0.23	0.23	0.12	0.18	1.000	1.61	0.80	1.61	0.80	0.80	1.61	1.61	0.80	**1.21**
*O*. g*ratissimum*	Acetone	0.117	0.09	0.09	0.19	0.38	0.19	0.78	0.38	0.38	0.31	0.180	0.14	0.14	0.29	0.58	0.29	1.20	0.58	0.58	0.48
	Cold water	0.134	0.05	0.10	0.07	0.11	0.09	**1.91**	0.11	0.05	0.31	0.041	0.02	0.03	0.02	0.03	0.04	0.59	0.03	0.02	0.10
*A*. *wilkesiana*	Acetone	0.132	0.21	**4.40**	0.43	0.88	0.21	0.29	0.29	**4.40**	**1.39**	1.560	**2.52**	**52.00**	**5.03**	**10.40**	**2.52**	**3.39**	**3.39**	**52.00**	**16.41**
	Cold water	0.070	**1.00**	0.06	0.11	0.06	0.03	0.06	0.03	0.03	0.17	0.470	**6.71**	0.38	0.71	0.38	0.19	0.38	0.19	0.19	**1.14**
Doxorubicin	-	0.022	-	-	-	-	-	-	-	-	-	0.0004	-	-	-	-	-	-	-	-	-

*Sa, Staphylococcus aureus; Ef, Enterococcus faecalis; Se, Salmonella Enteritidis; Sg, Salmonella Gallinarum; Ec (T), Escherichia coli (ATCC); Ec (Iso), Escherichia coli (Isolate); Cc, Campylobacter coli; Cj, Campylobacter jejuni; LC_50_ ≤ 0.03 mg/ml is toxic (American National Cancer Institute NCI). Selectivity Index values > 1 regarded to be more toxic to bacterial cells than mammalian cells (Bold)*.

The average SI values against Vero and Caco-2 cells for both bacterial and fungal organisms ranged from 0.01 to 4.48 and 0.005 to 16.41, respectively (Tables 7, 8). The cold water extract of *A*. *laxiflora* had the highest SI of 26.33 against *E*. *coli* isolates for Vero cells. The acetone extract of *A*. *wilkesiana* had the highest SI of 52 against *C*. *jejuni* for Caco-2 cells. The SI of *M*. *lucida* (cold water) against Vero and Caco-2 cells for *E*. *coli* isolates were 11.10 and 19.33, respectively (Tables 7, 8). The higher the SI the safer the plant extracts are potentially, but this has to be confirmed using *in vivo* tests.

**Table 8:** Cytotoxicity (LC_50_ in mg/ml) and selectivity index (SI) of plant extracts and selectivity index against Vero kidney and Caco-2 cells.

**Plant species**	**Extracts**	**Selectivity index**																	
		**Vero cells**									**Caco-2 cells**								
		**LC50**	***A***. ***fumigatus***		***A***. ***flavus***		***C***. ***albicans***		**Average**		**LC50**	***A***. ***fumigatus***		***A***. ***flavus***		***C***. ***albicans***		**Average**	
			**48 h**	**72 h**	**48 h**	**72 h**	**48 h**	**72 h**	**48 h**	**72 h**		**48 h**	**72 h**	**48 h**	**72 h**	**48 h**	**72 h**	**48 h**	**72 h**
*A*. *laxiflora*	Acetone	0.026	0.02	0.02	0.04	0.06	0.02	0.03	0.03	0.04	0.026	0.02	0.02	0.04	0.06	0.02	0.03	0.03	0.04
	Cold water	0.709	**2.29**	**2.29**	0.28	0.28	0.28	0.28	0.95	0.95	0.417	**1.35**	**1.35**	0.17	0.17	0.17	0.17	0.56	0.56
*F*. *exasperate*	Acetone	0.099	0.23	0.04	0.33	0.43	0.04	0.04	0.20	0.17	0.561	**1.22**	0.22	**1.87**	**2.44**	0.22	0.22	**1.10**	0.96
	Cold water	0.164	0.26	0.26	**1.09**	**1.09**	0.26	0.26	0.54	0.54	0.575	0.93	0.93	**3.83**	**3.83**	0.93	0.93	**1.90**	**1.90**
*M*. *lucida*	Acetone	0.033	0.02	0.02	0.22	0.17	0.07	0.07	0.10	0.09	0.041	0.03	0.03	0.27	0.22	0.09	0.09	0.13	0.11
	Cold water	0.333	2.22	2.22	**1.07**	**11.10**	0.13	0.13	**1.14**	**4.48**	0.580	**3.87**	**3.87**	**1.87**	**19.33**	0.23	0.23	**1.99**	**7.81**
*J*. *gossypiifolia*	Acetone	0.023	0.01	0.01	0.04	0.02	0.02	0.02	0.02	0.01	0.003	0.01	0.01	0.004	0.003	0.003	0.002	0.006	0.005
	Cold water	0.144	0.46	0.06	0.96	0.96	0.06	0.12	0.49	0.38	1.000	3.23	0.40	6.66	6.66	0.40	0.80	**3.43**	**2.62**
*O*. *gratissimum*	Acetone	0.117	0.05	0.05	3.90	0.78	0.09	0.09	**1.35**	**0.31**	0.180	0.07	0.07	**6.00**	**1.20**	0.14	0.14	**2.07**	0.07
	Cold water	0.134	0.10	0.22	0.43	0.43	0.09	0.07	0.21	0.24	0.041	0.03	0.07	0.13	0.13	0.03	0.02	0.06	0.07
*A*. *wilkesiana*	Acetone	0.132	**1.89**	0.21	0.11	0.21	0.88	0.88	0.96	0.43	1.560	**22.29**	**2.52**	**1.25**	**2.52**	**10.40**	**10.40**	**11.31**	**5.15**
	Cold water	0.070	0.03	0.03	0.03	0.03	0.03	0.03	0.03	0.03	0.470	0.19	0.19	0.19	0.19	0.19	0.19	0.19	0.19
Doxorubicin	-	0.022	-	-	-	-	-	-	-	-	0.0004	-	-	-	-	-	-	-	-

*A. fumigatus, Aspergillus fumigatus; A. flavus, Aspergillus flavus; C. albicans, Candida albicans. LC_50_ ≤ 0.03 mg/ml is regarded as toxic (American National Cancer Institute NCI). Selectivity index > shows selective toxicity to fungal cells than to mammalian cells (Bold)*.

## Discussion

### Antimicrobial Activity

In general, cold water offered the best yield of plant material while acetone rendered the lowest amount of plant constituents. Generally, in this study, aqueous solvents offered the best yield in most of the tested plant species, but this does not necessarily translate to efficient extraction of antimicrobial substances. According to several authors, organic solvents like acetone remain better extractants of antimicrobial substances compared to other solvents like water (48). Acetone was noted to be the best extractant for screening and isolation of antimicrobial compounds from plants (49). This is because acetone has high capacity to extract compounds with a wide range of polarity. This does not imply that other solvents are not equally useful, as results obtained from extracts made with methanol, ethanol, and water were similarly promising.

There are no generally accepted standard MIC end-points for *in vitro* testing of plant extracts. However, Kuete (50), proposed that the antibacterial activity of a plant extract is considered significant when MIC values are below 0.1 mg/ml, moderate when 0.1 ≤ MIC ≤ 0.625 mg/ml and weak when MIC > 0.625 mg/ml. In this study, Gram-negative organisms (*Salmonella, Escherichia* and *Campylobacter* species) were more susceptible than Gram-positive organisms (*S*. *aureus* and *E*. *faecalis*). This is contrary to the general belief that Gram-positive organisms are more susceptible than Gram-negative ones because of their weaker and less complex cell wall (51). Furthermore, apart from their cell membrane permeability, the observed resistance by the Gram-positive organisms could be ascribed to genetic factors such as dissemination of resistant genes (52). Interestingly, the ATCC strains of tested pathogens were more resistant than the chicken isolates, while the most sensitive chicken isolates were *Salmonella* Gallinarum and *Escherichia coli* (isolate). The most susceptible of the tested pathogens was the *E*. *coli* isolate and according to Ogundare and Onifade (53), the inhibition of establishment of *E*. *coli* by methanol extract of *M*. *lucida in vitro* and *in vivo* using agar well diffusion method and albino rats respectively showed good antibacterial activity with 25 mg/ml of the extract inhibited *E*. *coli* with a zone of inhibition measuring 5 mm. In a similar manner, Ndukwe et al. (54) reported appreciable activity of the aqueous root extract of *M*. *lucida* against *S*. *aureus, B*. *subtilis, E*. *coli*, and *P*. *aeruginosa* at MIC <2.5 mg/ml using the agar dilution method.

Generally, *A*. *laxiflora, F*. *exasperata* and *M*. *lucida* had better antibacterial activity than *J*. *gossypiifolia, O*. *gratissimum*, and *A*. *wilkesiana* against all tested pathogens. In a similar study, Akinpelu et al. (18) found that the hydromethanolic leaf extract of *A*. *laxiflora* had some activity against some bacteria and fungi strains. Using different antimicrobial assays, the antimicrobial activities of different parts of *F*. *exasperata* and other *Ficus* species has been reported. Suresh et al. (55) reported good antibacterial activities of the bark extracts of *F*. *racemosa* against standard strains and clinical isolates using micro broth dilution. In addition, the ethanol extract of the leaf of *F*. *exasperata* has been reported to have inhibitory activity (300 mg/ml) against *E*. *coli* using the well diffusion assay (56). Likewise, the methanol extract of the bark of *F*. *religiosa* was active against enterotoxigenic *E*. *coli* using disc diffusion (57). These reports on the antimicrobial activities of *F*. *exasperata* support the results from this present study.

The aqueous extracts of the leaves of *O*. *gratissimum* contain substances with antibacterial properties (58) this is in conformity with some our findings. The benzene extract of *J*. *gossypifiola* has been reported to have maximum antibacterial activity (zone of inhibition 13.05 ± 0.02 mm) against *E*. *coli* and *B*. *subtilis* while minimal efficacy (zone of inhibition 2.04 ± 0.02 mm) was observed with the aqueous extract (59). It was earlier reported that the range of MIC of methanol, ethanol and aqueous extracts of *A*. *wilkesiana* against *E*. *coli, S*. *aureus, K*. *pneumoniae, S*. *typhi, B*. *cereus*, and *S*. *dysenteriae* is 10–30 μg/ml using broth dilution methods (60).

*Escherichia coli, Salmonella* and *Campylobacter* organisms are best-known among human intestinal microbial flora and are versatile gastrointestinal pathogens. Several *E*. *coli* strains that have been incriminated in the cause of diarrhea have a distinct mode of pathogenesis (61). Campylobacteriosis is commonly associated with eating raw or undercooked poultry. *Escherichia coli* is the most common cause of bacterial diarrhea, affecting an estimated 2.4 million people each year in the United States (62). Our findings in this study showed the potential of the selected plants as a good candidate for testing against a wide range of diarrhea-causing chicken bacterial diseases.

With regard to antifungal activity, according to Aligiannis et al. (63) and Hamza et al. (64) MIC values of 0.5 mg/ml or less are considered to be significantly active, moderate with MIC between 0.6 and 1.5 mg/ml and weak with MIC above 1.6 mg/ml. In this study, *A*. *flavus* was the most susceptible fungus to most extracts of the six plants studied at 48 and 72 h incubation. Acetone and cold water extracts of all the plants displayed the best antifungal activity against at least one or more tested fungi, and are most likely to be generally fungistatic as growth of the pathogens appeared to resume after 72 h of incubation with INT (Table 2).

However, *A*. *flavus* and *C*. *albicans* were more susceptible to these extracts at the longer incubation period (72 h) so whether a longer contact period with fungal pathogens can potentiate bioactive constituents of the plants is a subject of further studies. The aqueous extract of *M*. *lucida* had excellent antifungal activity against all the tested chicken fungi this is similar to findings from previous studies by Banerjee et al. (65) and Jainkittivong et al. (66) who reported the morphological conversion of *C*. *albicans* and the germination of *Aspergillus nidulans* by the water-soluble components in *Morinda citrifolia* (Noni) which made it an option for anti-fungal therapy for candidiasis and aspergillosis in humans. Likewise, good antifungal activity of alizarin−1- methyl ether, anthraquinone isolated from dichloromethane extract of the roots of *M*. *lucida* against *Aspergillus fumigatus* and *Trichophyton mentagrophytes* at MIC values of 100 and 50 μg/ml, respectively has been reported (67).

The total antimicrobial activity indicates the volume to which the amount extracted from 1 g of the plant can be diluted with retention of activity. The higher the total activity of a plant extract, the more potent it is and the higher its usefulness and economic value (40). In this study, the significantly higher average total activity against bacterial pathogens than fungal pathogens indicated better potency against bacterial than against fungi.

The results from the present study showed *in vitro* antimicrobial activity of selected plant species as a possible candidate for testing against bacterial and fungal pathogens implicated in causing infectious diseases in poultry. However, *in vivo* data is necessary in future to determine the potential usefulness of these plants for management of infectious diseases as factors such as absorption, metabolism and enzymatic activation influence *in vivo* efficacy (68).

### Bioautography

In the bioautography analysis, most of the antimicrobial compounds were visible in the extracts prepared using polar solvents, and *S*. *aureus* was the most susceptible organism. All the extracts had weak activity against the fungi. *J*. *gossypiifolia* had the highest number of bands indicating presence of active compounds against all the tested organisms while *A*. *wilkesiana* had the lowest number of bands. The observed antibacterial activity of *J*. *gossypiifolia* may be attributed to general toxicity to both animal and bacterial cells as indicated by its toxicity to both Vero and Caco-2 cells, therefore care should be exercised in its use as a feed additive or it can be suggested for external use, although cautiously. In this study, little to no activity observed in some crude plant extracts may be ascribed to very low concentrations of the active compounds in the crude plant extracts (69). Furthermore, all the test plant extracts had fewer zones of inhibition against *A*. *fumigatus* and *C*. *albicans*. However, absence of activity could be attributed to factors including evaporation of active compounds, photo-oxidation or a low quantity of active compound.

Generally, the acetone extracts of the six plant species had good to moderate activity against both bacterial and fungal organisms. The cold water extracts of the six plant species had weak activity against the tested bacterial organisms but better antifungal activity with very low toxicity to both mammalian cell lines compared to other extracts. The aqueous extracts (cold water) are more relevant to the clinical application of the powdered leaves as alternative feed additives. The bioactive constituents of the feed additives will be released into the chicken gut fluids before final absorption into the general circulation. The low antimicrobial activity of the aqueous extracts may be attributed to their inability to extract the bioactive compounds in the plants compared to acetone (48). It is also possible that antimicrobial effects of these aqueous extracts are not mediated through direct inhibition on microbial growth but rather through immunostimulation, or the bioactive compounds may need metabolic activation by certain enzymes *in vivo*. The choice of aqueous (cold water) extracts agreed with the traditional applications of these plant species as antimicrobials coupling with their good and overall antimicrobial potentials alongside with the replication of the possible safety of mixtures of the plant powder with biological fluids (chicken gut fluids). Also, it was observed that cold water produced the best yield from the extraction process. In order to fulfill the main objective of the overall study to which these experiments contribute, which is the production of feed additives from powdered leaves, the aqueous extracts were chosen for further studies.

### Anti-biofilm Activity

Aqueous extracts of the majority of plant species tested in this study had good ABF activity (>50% inhibition) against *C*. *jejuni* compared to that of acetone extracts. Good inhibitory activity (>50% inhibition) against planktonic cells of *E*. *coli, C*. *coli, C*. *jejuni*, and *S*. Gallinarum was exhibited by most of the extracts at T_0_ indicating that prevention of biofilm attachment and growth proved to be easier to achieve than inhibition of pre-formed biofilms (T_24_) because the cells at T_0_ are not fully attached compared to those of T_24_.

Similarly, Mohsenipour and Hassanshahian (43), while evaluating ABF activity of the alcoholic extract of *A*. *sativum* against *E*. *coli* observed higher values (%) in the inhibition of biofilm formation than the values (%) for the destruction of already formed biofilm. In this study, all extracts and fractions enhanced the formation of biofilms of *C*. *coli* and *S*. Gallinarum and were expressed as 0% inhibition. This promotion of biofilm growth could be attributed to the presence of metabolites or production of conditioning films for microbial adhesion that may enhance the growth and development of biofilms (42). Sandasi et al. (42) made a similar observation in their investigation of the ABF activity of selected herbs, spices and beverages against *Listeria monocytogenes*. Furthermore, the presence of an EPS (glycocalyx) and negative charge on the EPS are among factors that have been linked to the ability of pathogens to form biofilms, while the negative charge limits the infiltration of molecules by charge attraction, thus causing resistance (70). In addition, plant lectins have been reported to improve the adsorption of cells onto a surface by acting as receptors of bacterial glycan, thereby enhancing cell attachment (71). Resistance and persistence of *Salmonella* has been attributed to their ability to form biofilms in abiotic surfaces outside the host, such as in farms, the food processing industry, kitchens or toilets, on plant surfaces, or even in animal epithelial cells (72).

In view of the role of the *Salmonella* genus in antimicrobial resistance (AMR), the anti-biofilm potential of extracts of *M*. *lucida* were further evaluated against eight *Salmonella* serovars that are relevant in livestock infections. Out of all the *Salmonella* serovars tested, the best ABF activities (>50% inhibition) were observed against *Salmonella* Enteritidis by the acetone extract of *M*. *lucida*, which may be attributed to the differential solubility of the bioactive compounds with solvent polarity (73). Good inhibitory activity (>50% inhibition) was exhibited against at least four of the *Salmonella* serovars by acetone and aqueous extracts of *M*. *lucida*, which is similar to the findings of Vijayan et al. (74) on the ability of the aqueous extract of another plant, *T*. *conoides* in the prevention of biofilm formation.

### Cytotoxicity and Selectivity Index

Acetone and cold water extracts of the six plant species were tested against Vero kidney and Caco-2 cell lines for cytotoxicity. According to Makhafola et al. (75), no crude plant extracts or natural products are regarded as safe for use until they are subjected to cellular toxicity tests. It is imperative to determine the cytotoxicity of a plant extract by using more than one cell line because the establishment of the safety and usefulness of a plant extract using only one cell line might be misleading. It is expected that the sensitivity of the cell lines to the extracts will be different because of different metabolic activities and uptake capabilities (76). The human intestinal cell line (Caco-2) has been reported for its known uptake capabilities (77). Therefore, the choice of Caco-2 cells in addition to Vero cells was made owing to its uptake capabilities and ability to serve as an absorptive surface for the bioactive ingredients in the feed additives. A study comparing the toxin Cylindrospermopsin (CYN) toxic effects in four different cell lines indicated that Caco-2 cells were one of the most sensitive to the toxin (78).

In another study by Pinto (79), it was demonstrated that Caco-2 cells, upon differentiation, expressed several morphological and biochemical characteristics of small intestinal enterocytes. In order to reduce the use of experimental animals for toxicity testing, the Caco-2 cell model has been considered for the development of alternative *in vitro* toxicity tests. In addition, the gastrointestinal tract is relevant for the absorption and biotransformation of xenobiotics due to the extensive area of exposure to orally ingested drugs, feed additives and contaminants. Moreover, the gastrointestinal tract can be a direct target for several toxicants (80). The extracts with LC_50_ > 0.1 mg/ml are considered to have negligible cytotoxicity (50). Also, the American National Cancer Institute (NCI) proposed that crude extracts are highly cytotoxic at LC_50_ ≤ 0.03 mg/ml following incubation with cells between 48 and 72 h (81).

The aqueous extract of *A*. *laxiflora* and *M*. *lucida* had the highest LC_50_ values (lowest toxicity) against Vero and Caco-2 cell lines respectively while the acetone extract of *J*. *gossypiifolia* had the lowest LC_50_ values (most toxic) against Vero and Caco-2 cell lines. Therefore, the antimicrobial activity of the *J*. *gossypiifolia* might be attributed to general toxicity. The antiproliferative activity of the aqueous extract of *M*. *lucida* against human promyelocytic leukemia (HL-60) cell lines has been reported (82). Our findings in this study showed that all the tested extracts were relatively safe against both Vero and Caco-2 cell lines except for the acetone extract of *J*. *gossypiifolia*. This plant has been previously reported to be toxic although its toxic nature has been mostly associated with the latex and seeds (83).

The selectivity index (SI) expresses the correlation between the antimicrobial and cytotoxic activities of the plant extracts on bacterial and normal cells so that the biological activity of the plant extract is not attributed to constituent toxic principles. Kudumela et al. (84) and McGaw et al. (85) reported that SI values greater or equal to 10 indicate a promising hit for product development, necessitating *in vivo* studies. Generally, SI above 1 is an indication that the biological activity of the plant extracts or natural products is higher than their cellular toxicity. The aqueous extract of *M*. *lucida* had the highest SI values against both Vero and Caco-2 cell lines therefore the aqueous extract of *M*. *lucida* was the safest of all the tested extracts in this *in vitro* study. Caco-2 cells were generally less susceptible than Vero cells to the tested extracts.

## Conclusion

The selected plant extracts had varying antimicrobial activity against relevant poultry bacteria and fungi which indicate the potentials of the plant species as a candidate for future testing *in vivo* in form of natural feed additives against relevant poultry pathogens. The cold water extract of *M*. *lucida* had the lowest MIC against *E*. *coli* (isolate) and *A*. *flavus*, respectively. Generally, the acetone extract of *M*. *lucida* exhibited the best ABF activities against *S*. Enteritidis while the aqueous extracts of same plant displayed good inhibitory activity (>50% inhibition) against at least four of the *Salmonella* serovars. Due to the promising activity of *Morinda lucida*, further study in an *in vivo* chicken feed trial as a potential candidate for development as a feed additive is recommended in future. The findings from this study will provide researchers and chicken farmers with useful information on the use of additives which are not only cost effective but also of herbal origin.

## Data Availability Statement

The raw data supporting the conclusions of this article will be made available by the authors, without undue reservation.

## Author Contributions

OO conducted the experimental work, analyzed the results, and wrote the manuscript. All authors revised and edited the manuscript. LM and IF supervised the research and edited the final version. LM provided funding and facilities and submitted the manuscript. All authors contributed to the article and approved the submitted version.

## Funding

The National Research Foundation (NRF) of South Africa is thanked for providing a PhD scholarship for OO and research funding to LM (NRF grant number 111945). IF acknowledges the University of Pretoria for a Postdoctoral Fellowship.

## Conflict of Interest

The authors declare that the research was conducted in the absence of any commercial or financial relationships that could be construed as a potential conflict of interest.

## Publisher's Note

All claims expressed in this article are solely those of the authors and do not necessarily represent those of their affiliated organizations, or those of the publisher, the editors and the reviewers. Any product that may be evaluated in this article, or claim that may be made by its manufacturer, is not guaranteed or endorsed by the publisher.
