# Supplementary material for: Antibacterial and Antibiofilm Activity of Selected Medicinal Plant Leaf Extracts Against Pathogens Implicated in Poultry Diseases
Source: Front Vet Sci. 2022 Mar 2;9:820304. doi: 10.3389/fvets.2022.820304 (PMC8926311; doi:10.3389/fvets.2022.820304)
Supplement: Supplementary file 1 [file Data_Sheet_1.pdf]

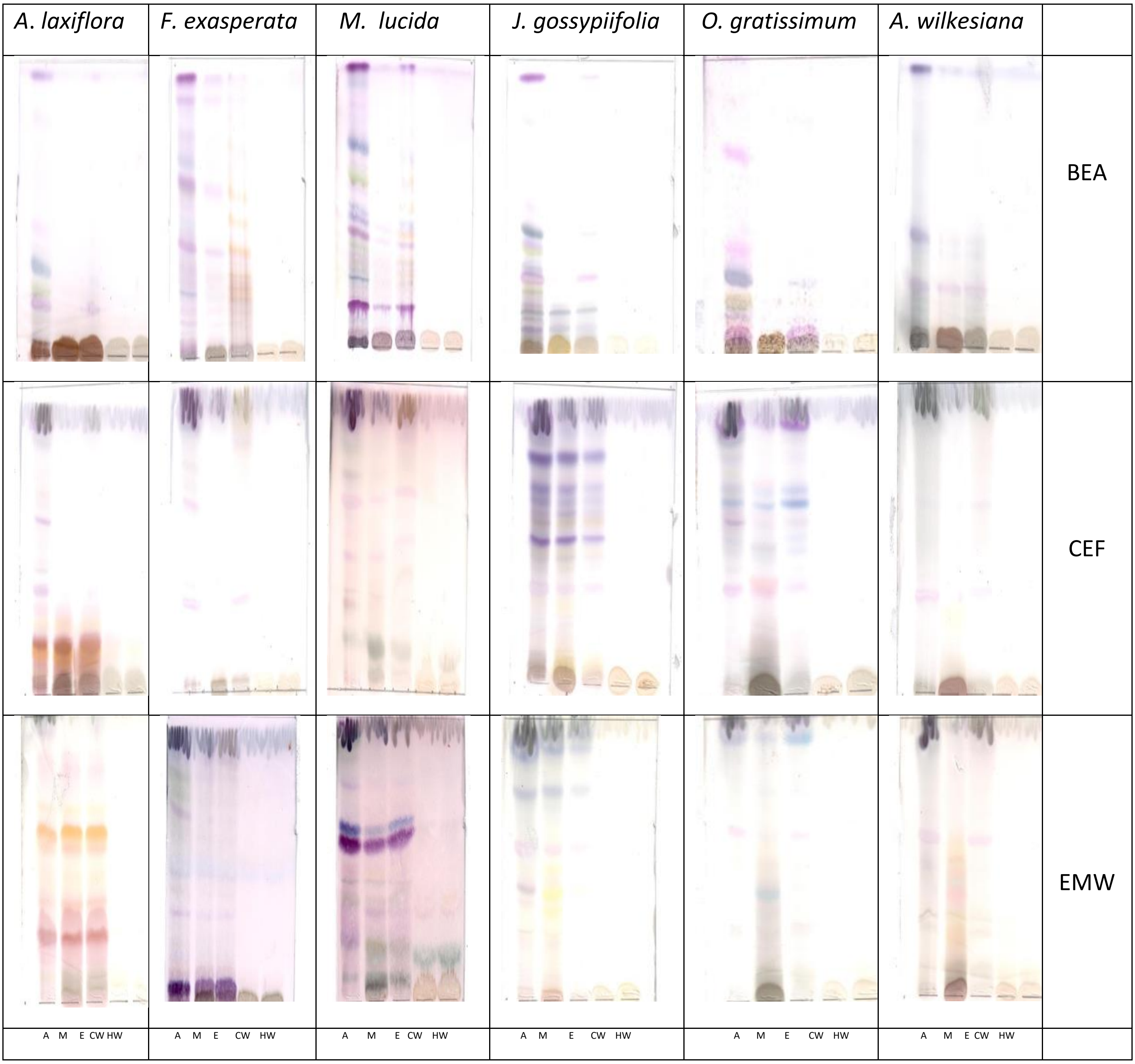

Fig. S1. Chromatographs of extracts of acetone (A), methanol (M), ethanol (E), cold water (CW) and hot water (HW) developed in Benzene: Ethanol: Ammonia hydroxide: 90:10:1 (BEA) (non-polar/basic), Chloroform: Ethyl acetate: Formic acid: 5:4:1 (CEF) (intermediate polar/acidic) and Ethyl acetate: Methanol: Water: 40:5:4.4 (EMW) (polar/neutral) and sprayed with vanillin in concentrated sulphuric acid.

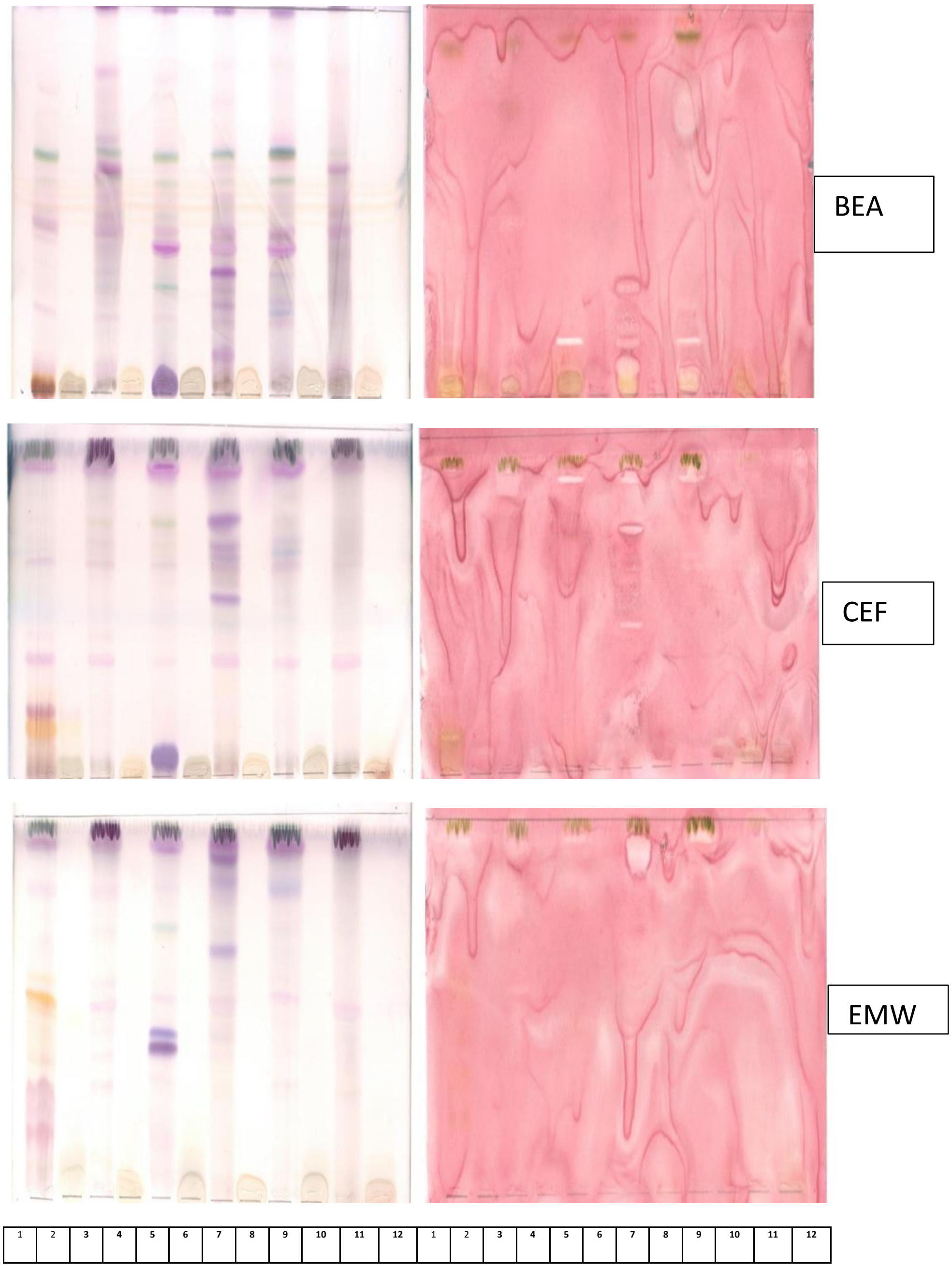

Fig S2. Chromatographs and bioautographs of acetone and aqueous extracts of **1** - *A. laxiflora* (acetone), **2** - *A.* l*axiflora* (aqueous), **3** - *F. exasperat*a (acetone), **4** - *F. exasperat*a (aqueous), **5 -** *M. lucida* (acetone), **6 -** *M. lucida* (aqueous), **7-** *J. gossypifiol*a (acetone), **8** - *J. gossypifiola* (aqueous), **9** - *O. gratissimum* (acetone), **10** - *O. gratissimum* (aqueous), **11**- *A. wilkesiana* (acetone) **12**- *A. wilkesiana* (aqueous) separated on TLC plates using EMW, BEA and CEF, sprayed with *E. coli* and 24 h later by INT. The white areas indicate zones of inhibition.

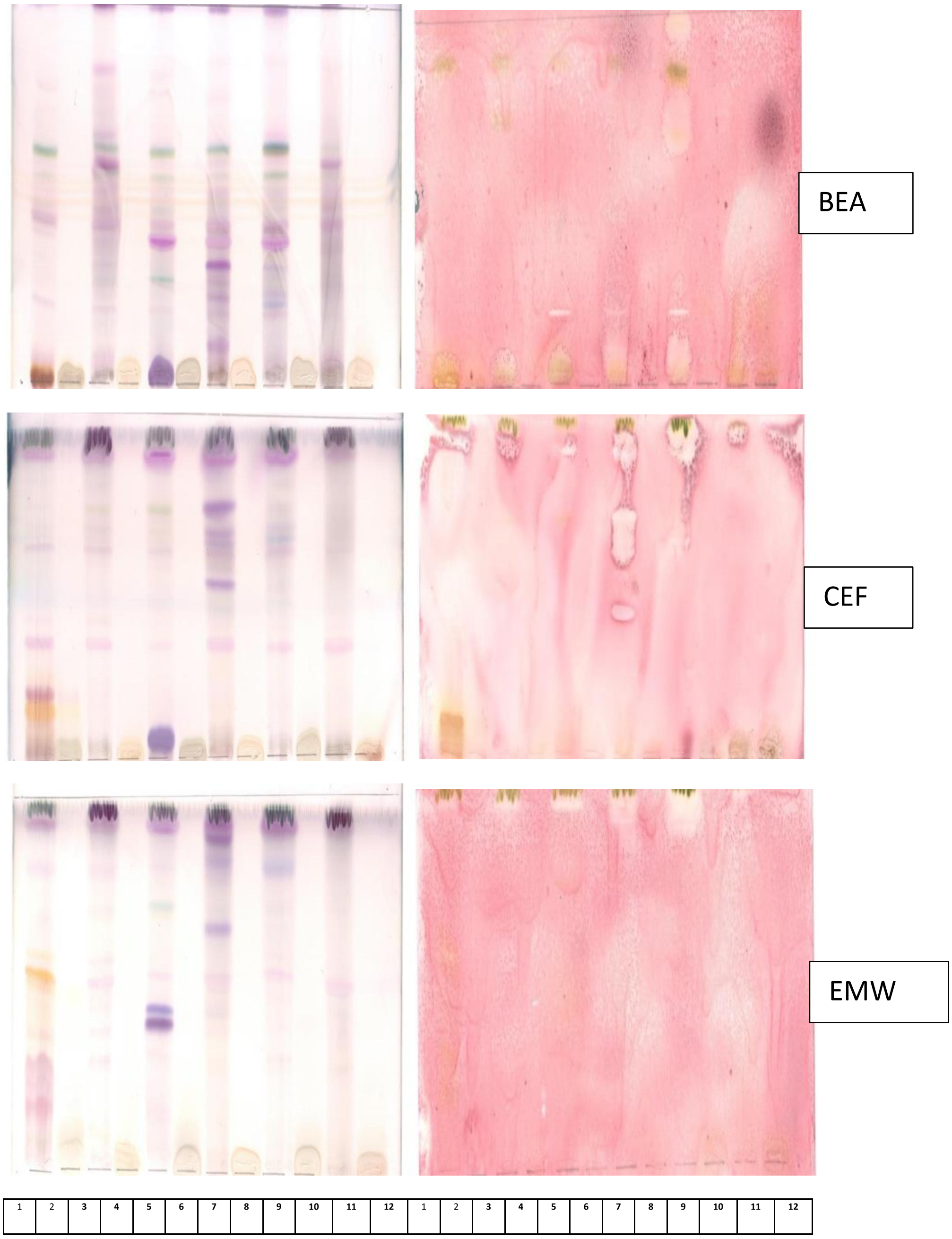

Fig S3 . Chromatographs and bioautographs of acetone and aqueous extracts of **1** - *A. laxiflora* (acetone), **2** - *A.* l*axiflora* (aqueous), **3** - *F. exasperata* (acetone), **4** - *F. exasperat*a (aqueous), **5 -** *M. lucida* (acetone), **6 -** *M. lucida* (aqueous), **7-** *J. gossypiifolia* (acetone), **8** - *J. gossypiifolia* (aqueous), **9** - *O. gratissimum* (acetone), **10** - *O. gratissimum* (aqueous), **11**- *A. wilkesiana* (acetone) **12**- *A. wilkesiana* (aqueous) separated on TLC plates using EMW, BEA and CEF, sprayed with *S. enteritidis* and 24 h later by INT. The white areas indicate zones of inhibition.

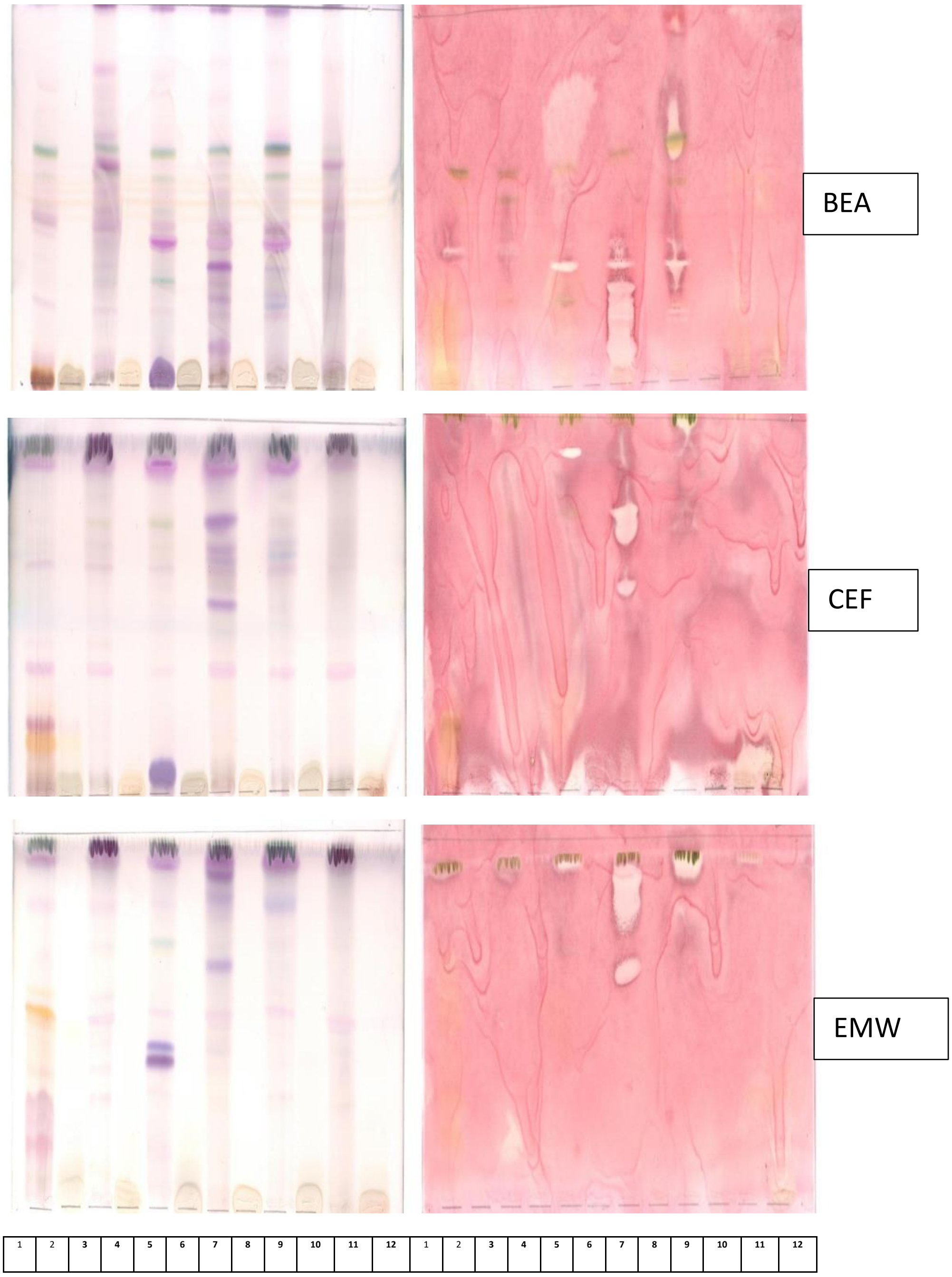

Fig S4. Chromatographs and bioautographs of acetone and aqueous extracts of **1** - *A. laxiflora* (acetone), **2** - *A. laxiflora* (aqueous), **3** - *F. exasperata* (acetone), **4** - *F. exasperata* (aqueous), **5 -** *M. lucida*(acetone), **6 -** *M. lucida* (aqueous), **7-** *J. gossypiifiola* (acetone), **8** - *J. gossypiifiola* (aqueous), **9** - *O. gratissimum* (acetone), **10** - *O. gratissimum* (aqueous), **11**- *A. wilkesiana* (acetone) **12**- *A. wilkesiana* (aqueous) separated on TLC plates using EMW, BEA and CEF, sprayed with *S. aureus* and 24 h later by INT. The white areas indicate zones of inhibition.

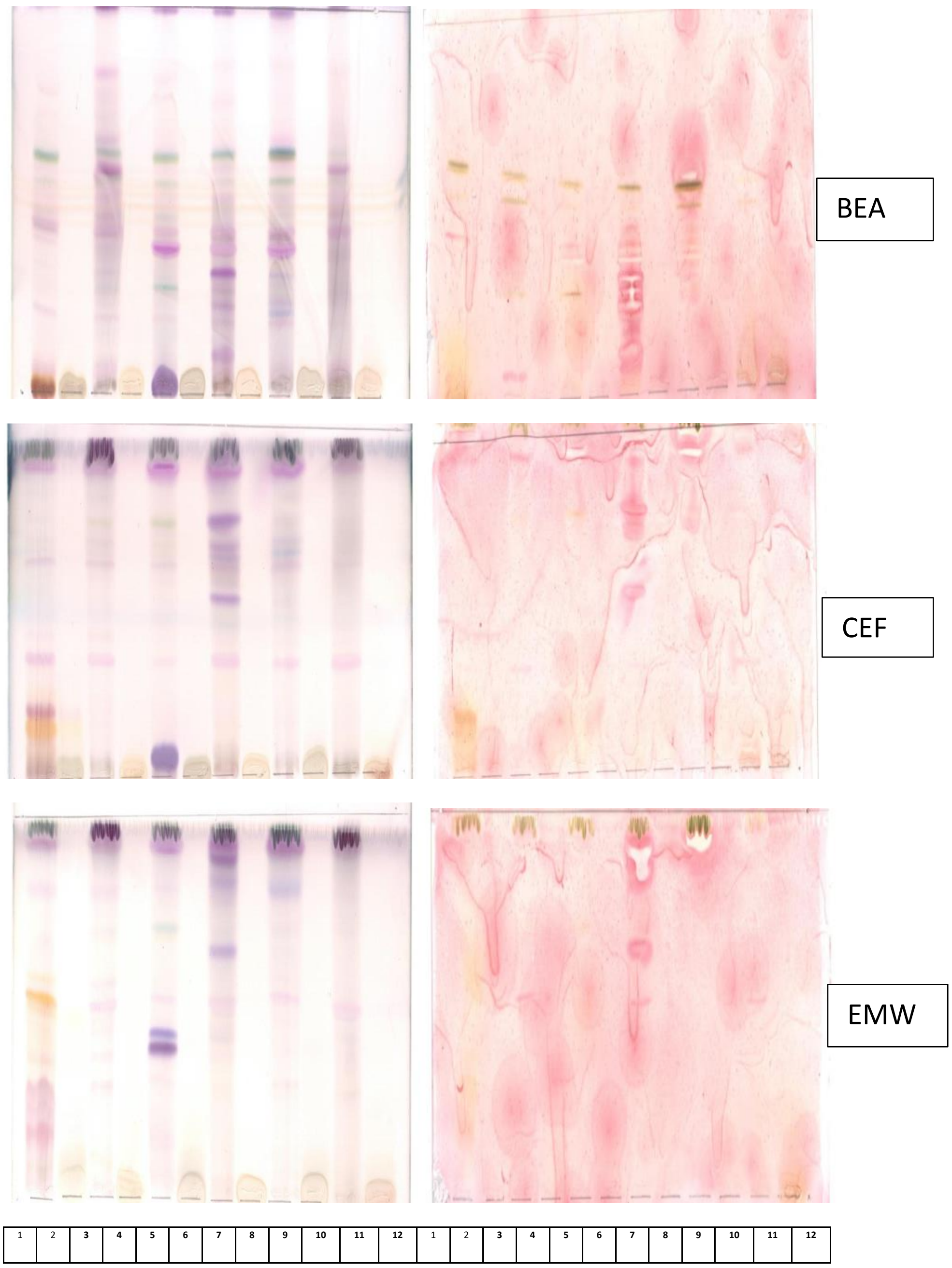

Fig S5. Chromatographs and bioautographs of acetone and aqueous extracts of **1** - *A. laxiflora* (acetone), **2** - *A.* l*axiflora* (aqueous), **3** - *F.* e*xasperata* (acetone), **4** - *F. exasperat*a (aqueous), **5 -** *M. lucida* (acetone), **6 -** *M. lucida* (aqueous), **7-** *J. gossypiifoli*a (acetone), **8** - *J. gossypifola* (aqueous), **9** - *O. gratissimum* (acetone), **10** - *O. gratissimum* (aqueous), **11**- *A. wilkesiana* (acetone) **12**- *A. wilkesiana* (aqueous) separated on TLC plates using EMW, BEA and CEF, sprayed with *C. jejuni* and 24 h later by INT. The white areas indicate zones of inhibition.

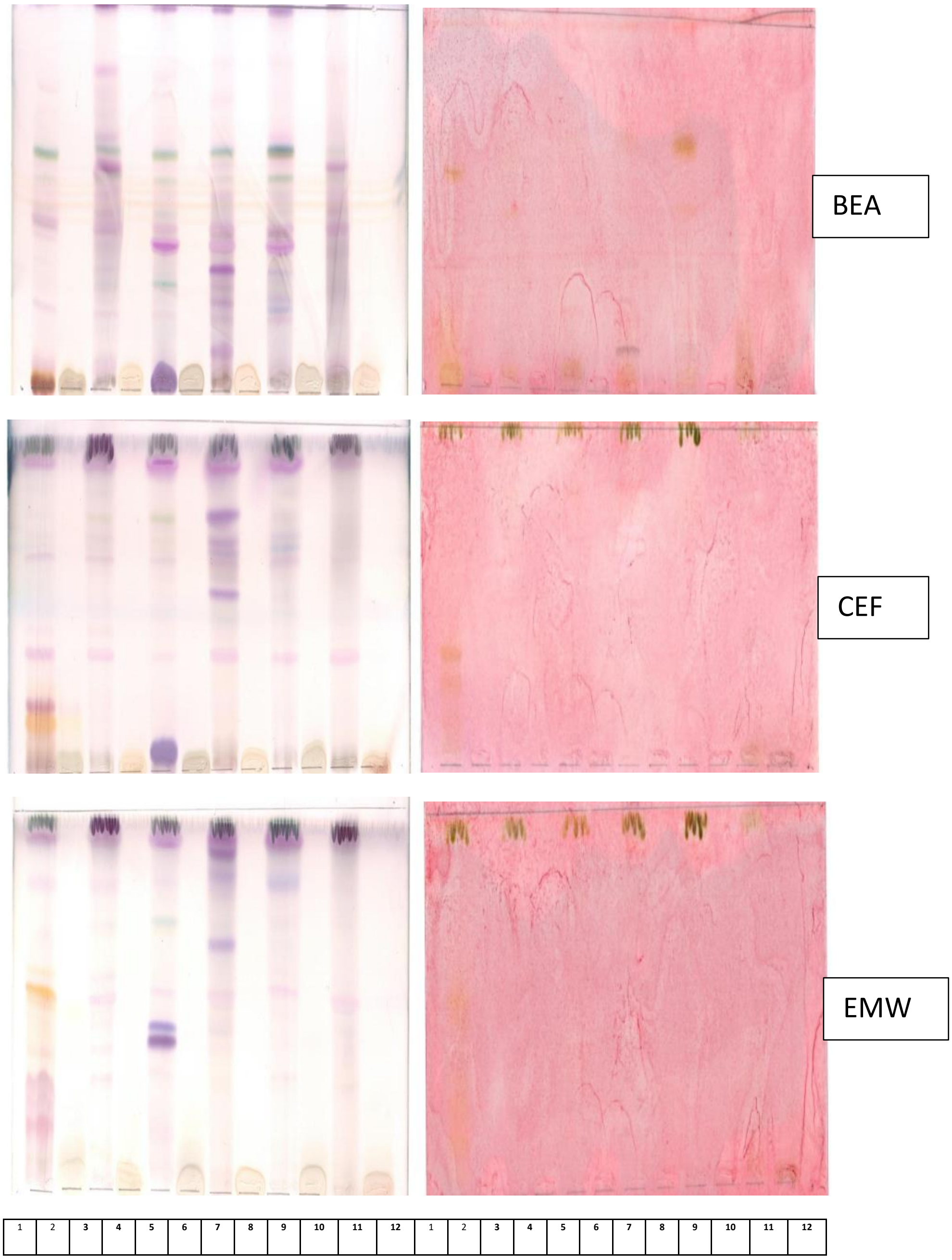

Fig S6. Chromatographs and bioautographs of acetone and aqueous extracts of **1** - *A. laxiflora* (acetone), **2** - *A. laxiflora* (aqueous), **3** - *F. exasperata* (acetone), **4** - *F. exasperata* (aqueous), **5** - *M. lucida* (acetone), **6** - *M. lucida* (aqueous), **7-** *J. gossypiifolia* (acetone), **8** - *J. gossypiifolia* (aqueous), **9** - *O. gratissimum*, (acetone), **10** - *O. gratissimum* (aqueous), **11**- *A. wilkesiana* (acetone) **12**- *A. wilkesiana* (aqueous) separated on TLC plates using EMW, BEA and CEF, sprayed with *A. fumigatus* and 24 h later by INT. The white areas indicate zones of inhibition.

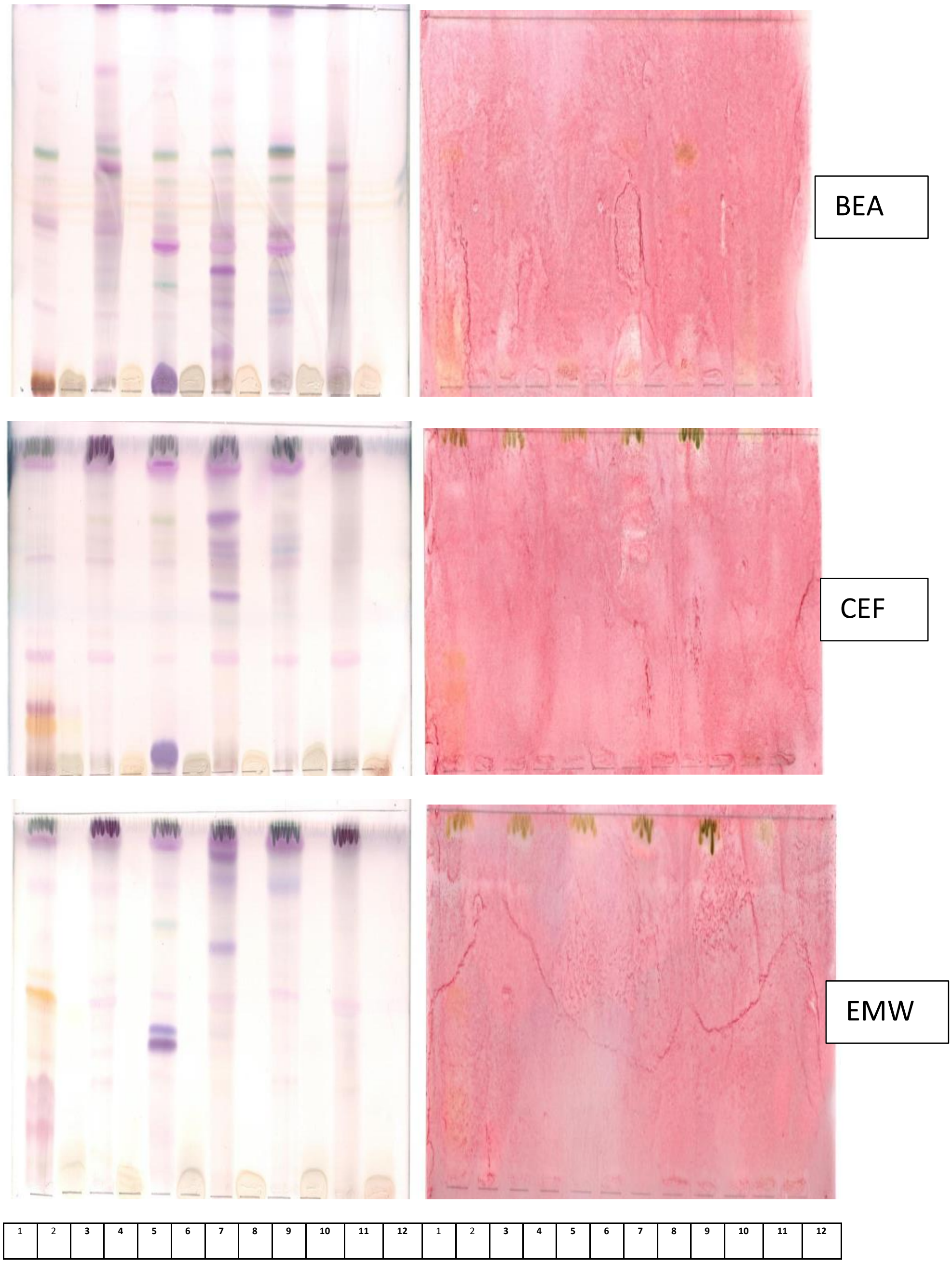

Fig S7. Chromatographs and bioautographs of acetone and aqueous extracts of **1** - *A. laxiflora* (acetone), **2** - *A.* l*axiflora* (aqueous), **3** - *F. exasperata* (acetone), **4** - *F. exasperat*a (aqueous), **5 -** *M. lucida* (acetone), **6 -** *M. lucida* (aqueous), **7-** *J. gossypiifoli*a (acetone), **8** - *J. gossypifiola* (aqueous), **9** - *O. gratissimum* (acetone), **10** - *O. gratissimum* (aqueous), **11**- *A. wilkesiana* (acetone) **12**- *A. wilkesiana* (aqueous) separated on TLC plates using EMW, BEA and CEF, sprayed with *C. albicans* and 24 h later by INT. The white areas indicate zones of inhibition.
